# Supplementary material for: Active transcription and Orc1 drive chromatin association of the AAA+ ATPase Pch2 during meiotic G2/prophase
Source: PLoS Genet. 2020 Jun 22;16(6):e1008905. doi: 10.1371/journal.pgen.1008905 (PMC7332104; doi:10.1371/journal.pgen.1008905)
Supplement: S1 Text — (DOCX) [file pgen.1008905.s001.docx]

**Active transcription and Orc1 drive chromatin association of the AAA+ ATPase Pch2 during meiotic G2/prophase**

Richard Cardoso da Silva, María Ascensión Villar-Fernández and Gerben Vader

**Supplementary data**

**Supplementary results and discussion**

A previous study reported artefactual ChIP enrichments in highly transcribed coding regions (HiperChIPable regions) [1]. We performed several analyses and experiments to ascertain that the binding patterns we found for Pch2 are not a result of such artefactual effects (see below). Based on these combined results we conclude that the Pch2 binding patterns observed here are a reflection of physiologically relevant effects.

1. We compared a “hyperChIPable” dataset obtained from yeast strains expressing GFP harboring a nuclear localization signal (NLS-GFP) [1] with our datasets. Peak calling was based on a highly stringent cut-off p-value of 10^-15^, a significance used by others when reporting meiois-specific ChIP-seq data sets [2]. Re-analysis of the reported NLS-GFP data set [1] using the same cut-off value revealed no significant peaks. NLS-GFP crosslinks non-specifically with >273 peaks, which often are at genes encoding for ribosomal factors and tRNA genes (these account for ~70% of the peaks) [1]. Our identified list of Pch2 binding sites showed little overlap with the hyperChIPable dataset: only 18 (RNAPolII-transcribed) of a total of 436 genes (*i.e.* ~4%) were reported within the NLS-GFP dataset (S3A Fig). Accordingly, we detected no enrichment for Pch2 at tRNAs genes (S3B Fig) and other non-coding RNAs, despite the fact that all the tRNA genes reported as artefactual ChIP signals are expressed during meiosis [3]. HyperChIPable regions show a positive correlation between ChIP signal and RNAPII activity [1]. In contrast, we detected a weak correlation between individual Pch2-binding sites and expression levels of the corresponding CDS (Pearson’s test, R^2^=0.3789, S2F Fig). Inspection of the 18 binding sites that overlap between our dataset and the NLS-GFP dataset also revealed that 75% of these possess relatively moderate Pch2-enrichment scores in our ChIP-seq dataset.
2. HyperChIPpable regions have been suggested to originate from non-physiological crosslinking artefacts by which (*i.e.* an “inert” nuclear GFP shows crosslink-based enrichment) [4]. Based on such a scenario, any nuclear factor should exhibit significant artefactual binding. We used a strain expressing 3XFLAG-dCas9 (without the co-expression of a guide (g)RNA), an inert nuclear protein − containing an NLS − that is not expected to bind any specific loci without a gRNA) to query association with a peak identified in our Pch2 dataset, using ChIP-qPCR, and compared it to enrichment as seen with 3XFLAG-Pch2 (S3C and 3D Fig). Although ChIP-qPCR readily detected a strong enrichment of 3XFLAG-Pch2 with this locus, 3XFLAG-dCas9 showed very minor enrichment, which was barely stronger than untagged controls.
3. We observed stronger enrichment in ChIP datasets from Pch2-E399Q as compared to wild type Pch2 (see above, and S2D Fig). The same effect was also observed in ChIP-qPCR experiments (for example see S2J Fig). We also used a mutant of Pch2, lacking its NTD to investigate binding, and we observed a strong dependency of Pch2 ChIP signals on this domain (S2J Fig). These experiments indicate that the association of Pch2 to chromatin behaves as would be expected, based on the known biochemical behavior of AAA+ enzymes [4]
4. We found that Pch2 binding depends on Orc1 function, and the BAH domain of Orc1 (Fig 4), something that would not be expected when caused by a specific crosslinking of a nuclear factor. Importantly, investigation of mRNA levels of two selected Pch2-associated genes showed that impairing Orc1 function (via *orc1-161*), which leads to loss of Pch2 recruitment, does not lead to a reduction of transcriptional activity of these genes (S6F Fig).
5. We corroborated our ChIP-based analysis using independent cytological analyses (immunofluorescence on meiotic chromosome spreads). These analyses demonstrate that in cells where *i)* transcription was inhibited (Fig 3) or *ii)* Orc1 function was impaired (via usage of *orc1-161*), Pch2 recruitment was impaired (Fig 4).
6. Ectopic expression of Pch2 in mitosis does not lead to recruitment of Pch2 to sites of active transcription (Fig 6). Thus, under conditions where the interrogated regions were transcriptionally active (and Orc1/ORC was present), Pch2 was not recruited to chromosomes.
7. Pch2 recruitment depends on Zip1 function (Fig 6), as judged by ChIP-based analysis. This is in agreement with earlier cytological studies that have established a role for Zip1 in driving the chromosome recruitment of Pch2 [5] (See also Fig 3I and S5H Fig).
8. We noted that *GAP1*, a gene that is highly expressed both in exponentially cycling cells and during meiosis was also identified as a site of hyperChIPpability (*i.e.* it is one of the 18 genes that shows an overlap between our Pch2 datasets and the NLS-GFP dataset) [1]. Importantly, in agreement with the results obtained by ChIP-seq, we confirmed the association of wild-type Pch2 and Pch2-E399Q to three additional selected binding genes (*HOP1*, *TDH3* and *SSA1*), none of which were present in the hyperChIPable dataset (Fig 1F and S2J Fig).

**Supplementary materials and methods**

**ChIP-qPCR primers**

Primer efficiencies (PE) were calculated using standard procedures.

pGV2390 5’-AGAACGTCATCTCCGGAATCT-3’ PE=1.998

pGV2391 5’-TGGGCACGATGAGAGAAAGT-3’

pGV2392 5’- GCGAAGAAGGGTTGGATGTT -3’ PE=1.961

pGV2393 5’- AGACGGTGCCATCGATGATA -3’

pGV2577 5’-AAGCTTTTCATCCCAGCAGA- 3’ PE=1.991

pGV2578 5’-TTTTTGTCGTTGTTCGATTCA- 3’

pGV2583 5’-ACCATCAGGACTGGAAGTGG- 3’ PE=2.006

pGV2584 5’-CCTGTGGTGACGAAGAATCA- 3’

pGV2587 5’-CTTGAACAGCAGCACCGTAA- 3’ PE=1.992

pGV2588 5’-TGGACCCAGTTGAAAAGGTC- 3’

pGV2593 5’-TGGTGGTACAAGAAGCGTTG- 3’ PE=1.990

pGV2594 5’-ACATCGCCATTGACTCCACT- 3’

pGV2595 5’-TCGAAGACCGAACAGGAACT- 3’ PE=1.953

pGV2596 5’-ACAATTGGTCCAAAGCATCC- 3’

pGV2597 5’-GCGGACAAGAATGTGTCTGA- 3’ PE=2.001

pGV2598 5’-TCTGCTGTAAATGGCAAACG- 3’

pGV2599 5’-GAGGACGAGCTGGTGGAATA- 3’ PE=1.962

pGV2600 5’-TTCTGTTTCAGGTCCCCAAG- 3’

pGV2605 5’-GGGGTTGTTCCCTGATGATA- 3’ PE=1.927

pGV2606 5’-CTCCCTTCTCCAACCAATCC- 3’

pGV2717 5’-CAAGAAATGCAAACCGCTGC-3’ PE=2.019

pGV2718 5’-GGTCAATACCGGCAGATTCC-3’

pGV2747 5’-GCCTTAGTAACGGCGAGTGA-3’ PE=1.976

pGV2748 5’-CACGGGATTCTCACCCTCTA-3’​

pGV2601 5’- ATTTCTGGCTGGCAGACTGT -3’ PE=1.788

pGV2605 5’- ATATGCGGAACCAAATCTCG -3’

pGV3195 5’- TGACCTCATGCAGCAAAGTC-3’ PE=1.936

pGV3196 5’- CCGGTTGCTCCAGAAGATAA-3’

pGV3197 5’- TCGCTCTGGATGAGACATA-3’ PE=2.147

pGV3198 5’- GTTGCTGTCCTTGGTCTG -3’

pGV3199’- TGAATCGAACAACGACAAAA-3’ PE=1.791

pGV3200 5’- GCACGGAAATATACAATTTTTGACTA -3’

pGV3201’- GAAGCTCACACAGATTAG-3’ PE=1.949

pGV3202 5’- GAAGCTCACACAGATTAG -3’

pGV3203’- GTATATAAAGACGGTAGG-3’ PE=2.003

pGV3204 5’- TATGTGTGTTTATTCGAA -3’

pGV3205’- GTTCATAGGTCCATTCTC-3’ PE=1.861

pGV3206 5’- CCAAATCAGAGAGAGCAG -3’

pGV3207’- TGATTGAAAGCTTTGTG-3’ PE=1.705

pGV3208 5’- AGCCAATATCCCCAAAAT -3’

pGV3209’- GTACAGCCATCAAATCATG-3’ PE=1.874

pGV3210 5’- CTTGGCGCGTACATTTAAT -3’

pGV3211 5’- ACCGCGATGAGCTTCATA-3’ PE=1.983

pGV3212 5’- AGGGTTACTCCTGTGCGA -3’

pGV3214 5’- AGGGTTACTCCTGTGCGA-3’ PE=2053

pGV3215 5’- CCTGAACCGATGACTTGA -3’

pGV3216 5’- GACAAATAATTCACTTCCTTA-3’ PE=2.004

pGV3217 5’- ACCAACGGCACTTGATACTTG -3’

pGV3218 5’- CGAAAGTCAAAGTGCCATACAA-3’ PE=2.157

pGV3219 5’- CAAGTATCAAGTGCCGTTGG -3’

**Yeast strains**

All strains, except yGV104 and yGV2941, which are of the W303 background, are derived from the SK1 background.

yGV**49** *MATa/MATalpha, ho::LYS2, lys2, ura3, leu2::hisG, his4B::LEU2, ARG4/*

*arg4-Bgl II*

yGV104 *MATa, ade2-1, leu2-3, ura3, trp1-1, his3-11,15, can1-100, GAL, psi+*

yGV933 *MATa/MATα, ho::LYS2, lys2, ura3, leu2::hisG, trp1::hisG, his3::hisG, his4B::LEU2, arg4-Bgl II, pch2::URA3:pPCH2(300bp):3HA-PCH2*

yGV1185    MATa/ MATalpha, ho::LYS2, lys2, ura3, leu2::hisG, TRP1, HIS3,  arg4-Bgl II, pch2::URA3:pPCH2(300bp):3HA-PCH2, orc1::orc1-161 (ts-allele)

yGV**1465**  *MATa/MATalpha,  ho::LYS2, lys2, ura3, leu2::hisG, his3::hisG, trp1::hisG*

*orc1::TRP1, ura3::orc1ΔNTD(1-235)::URA3*

yGV1506 *MATa/MATα, ho::LYS2, lys2, ura3, leu2::hisG, TRP1, HIS3,*

*his4B::LEU2, pch2::URA3:pPCH2(300bp):3HA-PCH2, orc1::ORC1-TAP::HIS3*

yGV1508 *MATa/MATα, ho::LYS2, lys2, ura3, leu2::hisG, HIS3, trp1::hisG, his4B::LEU2, orc2::ORC2-TAP::HIS3, pch2::URA3:pPCH2(300bp):3HA-PCH2*

yGV1786 *MATa/MATα, ho::LYS2, ura3, leu2::hisG, his3::hisG, his4X, trp1::hisG, ndt80Δ::TRP1, pch2::URA3:pPCH2(300bp):3HA-PCH2*

yGV1945 *MATa/MATα, ho::LYS2, lys2, ura3, leu2::hisG, HIS3, trp1::hisG, his4B::LEU2, orc2::ORC2-TAP::HIS3, pch2::URA3:pPCH2(300bp):3HA-PCH2, orc1::orc1-161*

yGV**2086** *MATa/MATalpha, ho::LYS2, lys2, ura3, leu2::hisG, TRP, HIS3*

*his4B::LEU2, arg4-Bgl II, pch2::URA3:pPCH2(300bp):3HA-PCH2-E399Q*

yGV2099 *MATa/MATα, ho::LYS2, lys2, ura3, leu2::hisG, trp1::hisG, his3::hisG, arg4- Nsp/arg4, his4X::LEU2-(BGV)-URA3, pch2Δ::KanMX, orc1::ORC1-TAP::HIS3*

yGV2234 *MATa/MATalpha, ho::LYS2, lys2, ura3, leu2::hisG, TRP1, HIS3*

*his4X, ARG4/ arg4-BglII, pch2::URA3:pPCH2(300bp):3HA- PCH2,  ndt80Δ::TRP1*

yGV2249 *MATa/MATα, ho:LYS2, lys2, ura3, leu2::hisG, TRP1, ARG4 ndt80Δ::TRP1, pch2::URA3:pPCH2(300bp):3HA-pch2-E399Q*

yGV2447 *MATa/MATα, ho::LYS2, lys2, ura3, leu2::hisG, his3::hisG, trp1::hisG, his4X::LEU2- URA3, trp1::pPch2::TRP1, ARG4, pch2Δ::KanMX, ndt80Δ::LEU2*

yGV**2484** *MATa/MATα, lys2, ho::LYS2, trp1::hisG, his3::hisG, leu2::hisG,ura3, rad17::LEU2*

yGV**2485** *MATa/MATα, lys2, ho::LYS2, trp1::hisG, his3::hisG, leu2::hisG, ura3*

*rad17::LEU2*

yGV**2487** *MATa/MATα, ho::LYS2, lys2, ura3, leu2::hisG, his3::hisG, trp1::hisG, trp1::pPch2::TRP1, pch2Δ::KanMX, rad17::LEU2*

yGV**2494** *MATa/MATα, ho::LYS2, lys2, ura3, leu2::hisG, ARG4, his3::hisG, ndt80Δ::TRP1, pch2::URA3:pPCH2(300bp):3HA-PCH2, zip1Δ::LYS2*

yGV**2498** *MATa/MATα, ho::LYS2, ura3, leu2::hisG, his3::hisG, trp1::hisG,*

*ndt80Δ::TRP1, pch2::URA3:pPCH2(300bp):3HA-PCH2, orc1::orc1-161 (ts-allele).*

yGV**2499** *MATa/MATα, ho::LYS2, lys2, ura3, leu2::hisG, his3::hisG, trp1::hisG, trp1::pPch2::TRP1, pch2Δ::KanMX, rad17::LEU2*

yGV**2799** *MATa/MATα, ho::LYS2, lys2, leu2::hisG, his3::hisG, trp1:pPCH2-3XFLAG-Pch2::TRP1, pch2Δ::KanMX, ARG4, his4X::LEU2-URA3*

yGV**2800** *MATa/MATα, ho::LYS2, lys2, leu2::hisG, his3::hisG, ura3, trp1:pPCH2- 3XFLAG-Pch2::TRP1, pch2Δ::KanMX, ARG4, rad17::LEU2*

yGV**2816** *MATa/MATα, ho::LYS2, lys2, leu2::hisG, his4X::LEU2-URA3, his3::hisG, ura3, trp1:pPCH2-3XFLAG-Pch2::TRP1, pch2Δ::KanMX, ARG4, orc1::ORC1-TAP::HIS3*

yGV2875 *MATa/MATα, ho::LYS2, lys2, leu2::hisG, his3::hisG, ura3, trp1:pPCH2-3XFLAG- Pch2-243-564::TRP1, pch2Δ::KanMX, ARG4, ndt80Δ::LEU2*

yGV2889 *MATa/MATα, ho::LYS2, lys2, leu2::hisG, his4X::LEU2-URA3, his3::hisG, ura3, trp1:pPCH2-3XFLAG-PCH2::TRP1, pch2Δ::KanMX, ARG4, ndt80Δ::TRP1*

yGV2899 *MATa/MATα, ho::LYS2, lys2, leu2::hisG, his3::hisG, ura3, trp1:pPCH2-3XFLAG- -PCH2::TRP1, pch2Δ::KanMX, ARG4, orc1::TRP1, ura3::orc1ΔNTD(1- 235)-TAP::HIS3::URA3*

yGV2919 *MATa/MATα, ho::LYS2, lys2, leu2::hisG, his4X::LEU2-URA3, HIS3 , ura3, trp1::hisG, pch2Δ::KanMX, ARG4, trp1:pPch2-3XFLAG-pch2 E399Q::TRP1, ndt80Δ::TRP1*

yGV2941 *MATa, ade2-1, leu2-3, ura3, trp1-1, his3-11,15, can1-100, GAL, psi+, ura3:pGAL10-3HA-pch2-E399Q::URA3*

yGV2950 *MATa/MATalpha, ho::LYS2, lys2, ura3, leu2::hisG, his3::hisG, trp1::hisG, ARG4, trp::p11_pHOP1_3xFlag-dCas9::TRP1*

yGV3304 *MATa/MATα, ho::LYS2, lys2, leu2::hisG, his4X::LEU2-URA3, HIS3, ARG4, ura3, trp1::hisG, pch2Δ::KanMX, ARG4, trp1:pPch2-3XFLAG-pch2 E399Q::TRP1, ndt80Δ::TRP1, orc1::orc1-161*

YGV3320 *MATa/MATα, ho::LYS2, lys2, ura3, leu2::hisG, his3::hisG, trp1::hisG, his4X::LEU2, trp1::pPch2::TRP1, ARG4, pch2Δ::KanMX, ndt80Δ::LEU2, orc1::orc1-161*

yGV3384 *MATa/MATα, ho::LYS2, lys2, ura3, leu2::hisG, TRP, arg4-Bgl II*

*pch2::URA3:pPCH2(300bp):3HA-pch2-E399Q, orc1::TRP1, ura3::ORC1 TAP::HIS::URA3*

yGV3422 *MATa/MATα, ho::LYS2, lys2, leu2::hisG, his4X::LEU2-URA3, HIS3, ura3, trp1::hisG, pch2Δ::KanMX, ARG4, trp1:pPch2-3XFLAG-pch2 E399Q::TRP1, ndt80Δ::TRP1, zip1Δ::NatMX4*

yGV3476 *MATa/MATα, ho::LYS2, lys2, ura3, leu2::hisG, his3::hisG, trp1::hisG, ndt80::NatMX4,  RPL13A-2XFKBP12::TRP1, fpr1::KanMX4, tor1-1::HIS3,*

*pch2::URA3:pPCH2(300bp):3HA-PCH2*

​

yGV3898 *MATa/MATα, ho::LYS2, lys2, ura3, leu2::hisG, his3::hisG, trp1::hisG, RPL13A- 2XFKBP12::TRP1, fpr1 Δ::KanMX4, tor1-1::HIS3, ndt80Δ::NatMX4, pch2::URA3:pPCH2(300bp):3HA-PCH2*

yGV3943 *MATa/MATα, ho::LYS2, lys2, ura3, leu2::hisG, his3::hisG, trp1::hisG, RPO21- FRB::KANMX6, RPL13A-2XFKBP12::TRP1, fpr1::KanMX4, tor1-1::HIS3, ndt80Δ::NatMX4, pch2::URA3:pPCH2(300bp):3HA-PCH2*

yGV4034 *MATa/MATα, ho::LYS2, lys2, ura3, his4B::LEU2, ARG4, pch2::URA3:pPCH2(300bp):3HA-PCH2-E399Q, orc2-1, ndt80Δ::NatMX4*

yGV4041 *MATa/MATα, ho::LYS2, lys2, ura3, TRP1, ARG4, orc2-1, ndt80Δ::NatMX4*

**yGV4161** *MATa/ MATalpha, ho::LYS2, lys2, ura3, leu2::hisG, TRP1, ARG4, pch2::URA3:pPCH2(300bp):3HA-pch2-E399Q****,****orc1::TRP1, ura3::orc1ΔNTD(1-235)::URA3*

**Yeast strains used per figure:**

1B: yGV2447, yGV2889 and yGV2919

1C: yGV2889 and yGV2919

1D: yGV2889

1E: yGV2889 and yGV2919

1F: yGV2447 and yGV2919

2A: yGV2889

2B: yGV2889 and yGV2919

3B: yGV3476 and yGV3898

3D: yGV3943

3E-G: yGV3898 and yGV3943

3H: yGV3943

3I: yGV3943, yGV2494, and yGV2249

4B: yGV2889 and yGV2919

4C: yGV2447 and yGV2919

4D: yGV1506, yGV1508 and yGV2447

4E: yGV2447 and yGV2919, yGV3304 and yGV3320

4F and G: yGV1786 and yGV2498

4H: yGV49, yGV1465, yGV2086 and yGV4161

5A and B: yGV3943

5C: yGV3943

6B: yGV2941, yGV2086

6C and D: yGV49, yGV104, yGV2941, yGV2086

6E: yGV2447, yGV2919, yGV3598

**Yeast strains used per supplementary figure:**

S1A: yGV2799 amd yGV2816

S1B: yGV2484, yGV2485, yGV2487, yGV2494, yGV2499,

S1C yGV2889

S1D: yGV2889 and yGV2919

S1E: yGV2889

S2A-D: yGV2889 and yGV2919

S2E-F: yGV2889

S2G: yGV2447 and yGV2919

S2H: yGV2889

S2I and J: yGV2447, yGV2875, yGV2889 and yGV2919

S3A: yGV2889

S3B: yGV2889 and yGV2919

S3C and D: yGV49, yGV2447, yGV2889, and yGV2950

S4: yGV2889

S5B-D: yGV2447 and yGV2919

S5E: yGV2234, yGV3898 and yGV3943

S5F: yGV2249

S5G: yGV3943

S5H: yGV2494, and yGV2249

S5I: yGV3943

S6A: yGV2919

S6B: yGV2447, yGV2919, yGV3304 and yGV3320

S6C: yGV933, yGV1185, yGV1508 and yGV1945

S6D: yGV2919 and yGV3304

S6E-F: yGV1786 and yGV2498

S6G-H yGV49, yGV2086, yGV4034, yGV4041

S6I: yGV2086, yGV4034, yGV4041

S7A-C: yGV1786 and yGV2498

S7D: yGV49, yGV2086 and yGV4161

S7E: yGV49, yGV1465, yGV2086 and yGV4161

S8: yGV2889

S9A: yGV2447 and yGV2889

S9B: yGV3943 and yGV2447

S9C-D: yGV3943

S9F: yGV3943

S9G: yGV2498 and yGV2249

S9H: yGV2494, and yGV2249

S10A: yGV104 and yGV2941

S10B-D: yGV2447, yGV2919 and yGV3422

S11: yGV2889 and yGV2447

**References**

1. Teytelman L, Thurtle DM, Rine J, van Oudenaarden A. Highly expressed loci are vulnerable to misleading ChIP localization of multiple unrelated proteins. Proc Natl Acad Sci U S A. 2013;110(46):18602-7.

2. Sun X, Huang L, Markowitz TE, Blitzblau HG, Chen D, Klein F, et al. Transcription dynamically patterns the meiotic chromosome-axis interface. eLife. 2015;4.

3. Brar GA, Yassour M, Friedman N, Regev A, Ingolia NT, Weissman JS. High-resolution view of the yeast meiotic program revealed by ribosome profiling. Science. 2012;335(6068):552-7.

4. Hanson PI, Whiteheart SW. AAA+ proteins: have engine, will work. Nat Rev Mol Cell Biol. 2005;6(7):519-29.

5. San-Segundo PA, Roeder GS. Pch2 links chromatin silencing to meiotic checkpoint control. Cell. 1999;97(3):313-24.
