# Supplementary figures and images for: Active transcription and Orc1 drive chromatin association of the AAA+ ATPase Pch2 during meiotic G2/prophase
Source: PLoS Genet. 2020 Jun 22;16(6):e1008905. doi: 10.1371/journal.pgen.1008905 (PMC7332104; doi:10.1371/journal.pgen.1008905)

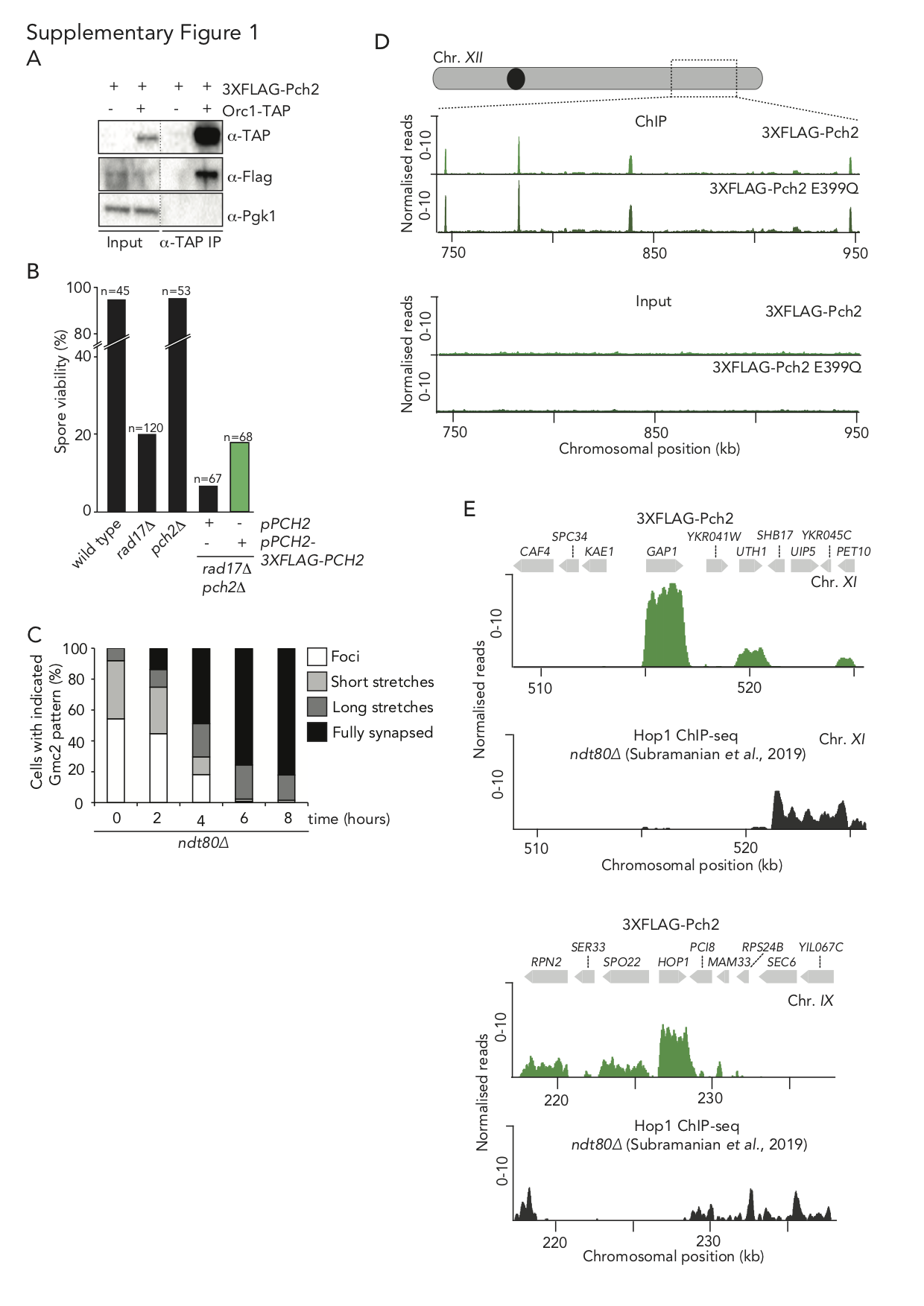

Supplement: S1 Fig — A. Co-immunoprecipation experiment showing an interaction between 3XFLAG-Pch2 and Orc1-TAP. Pgk1 was used as a control. B. Spore viability analysis of the indicated strains. Cells were sporulated for 24 hours in liquid media. The total analyzed tetrads/strain are indicated. C. Representation of meiotic G2/prophase progression in used strain. SC appearance was detected using Gcm2. For each time point 125 cells were counted. D. Genome browser view representative images (RPKM; see also Material and Methods) of ChIP and input signals for 3XFLAG-Pch2 and 3XFLAG-Pch2-E399Q. Shown is a region of Chromosome XII (chromosomal coordinates (kb) are indicated) identical to ChIP-seq binding patterns as shown in Fig 1C. E. High resolution Genome browser view representative images (RPKM; see also Material and Methods) of 3XFLAG-Pch2 and Hop1 binding patterns (from [44]) across two selected chromosomal regions (chromosomes XI and IX), identical to ChIP-seq binding patterns as shown in Fig 1D. Chromosomal coordinates and gene organization are indicated. (TIFF) [file pgen.1008905.s002.tiff]

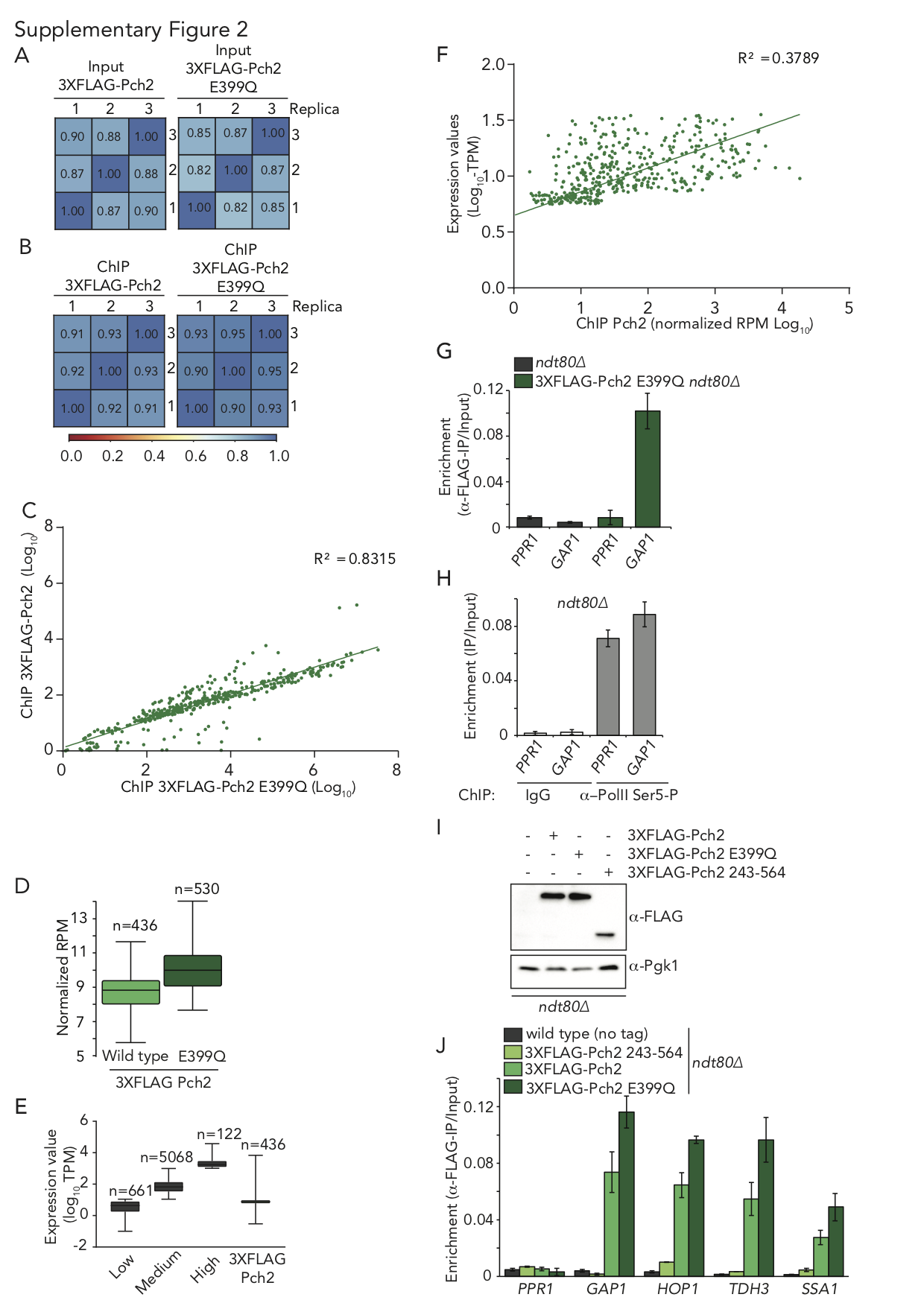

Supplement: S2 Fig — A and B. Heatmap matrices depicting correlation analysis for Pch2 ChIP-seq and Input replicates as measured by the Spearman correlation coefficient (R-values within the squares). C. Scatter plot correlation analysis of Pch2-ChIP-seq (Wild-type versus E399Q) normalized read counts (Log10—RPKM) measured by the Pearson correlation coefficient R-values (upper right corner). D. Normalized read counts (ChIP/input) for 3XFLAG-Pch2 and 3XFLAG-Pch2-E399Q during meiotic G2/prophase, as determined by ChIP-seq. E. Comparison between expression strength of Pch2-associated genes and the transcribed genes from our mRNA dataset (ndt80Δ cells) binned into high, medium and low expression strength (following previously established procedures [35]). F. Correlation between normalized reads (log10) of 3XFLAG-Pch2 individual binding sites and mRNA expression values (Transcription Per Million). G. ChIP-qPCR analysis of 3XFLAG-Pch2-E399Q at PPR1 (primer pair: GV2390/GV2391) and GAP1 (primer pair: GV2597/GV2598) during meiotic G2/prophase (4 hours). H. ChIP-qPCR analysis of active transcription (α-phosphoserine 5 Rpo21) at PPR1 (primer pair: GV2390/GV2391) and GAP1 (primer pair: GV2597/GV2598) during meiotic G2/prophase (t = 4 hours). I. Western blot analysis of expression of 3XFLAG-Pch2, 3XFLAG-Pch2-E399Q and 3XFLAG-ΔNTD-Pch2 during meiotic G2/prophase (t = 4 hours). J. ChIP-qPCR analysis of 3XFLAG-Pch2, 3XFLAG-Pch2-E399Q and 3XFLAG-ΔNTD-Pch2 at PPR1 (primer pair: GV2390/GV2391), GAP1 (primer pair: GV2597/GV2598), HOP1 (primer pair: GV2607/GV2608), TDH3 (primer pair: GV2591/GV2592), and SSA1 (primer pair: GV2587/GV2588) during meiotic G2/prophase (t = 4 hours). Error bars represent standard error of the mean of at least three biologically independent experiments performed in triplicate. (TIFF) [file pgen.1008905.s003.tiff]

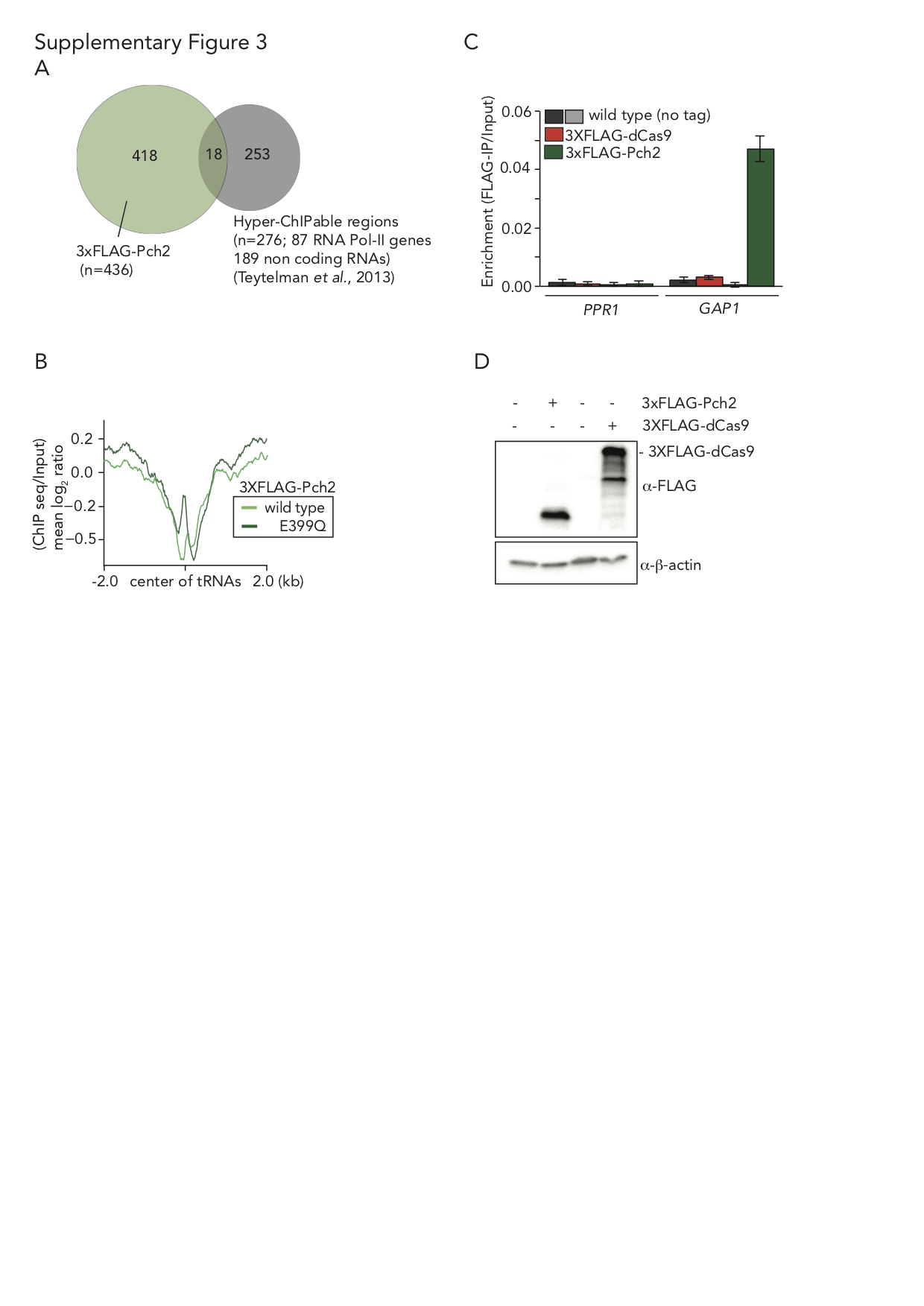

Supplement: S3 Fig — A. Venn diagram comparing 3XFLAG-Pch2 binding peaks and HyperChIPpable regions as described by [1]. B. 3XFLAG-Pch2 and 3XFLAG-Pch2-E399Q ChIP-seq normalized read counts enrichment normalized to inputs (log2). Datasets were aligned relative to centre of tRNAs. C. ChIP-qPCR analysis of 3XFLAG-Pch2 and 3XFLAG-dCas9 at PPR1 (primer pair: GV2390/GV2391) and GAP1 (primer pair: GV2597/GV2598) during meiotic G2/prophase (4 hours). Error bars represent standard error of the mean of at least three biologically independent experiments performed in triplicate. D. Western blot analysis of 3XFLAG-Pch2 and 3XFLAG-dCas9 as used in C. (TIFF) [file pgen.1008905.s004.tiff]

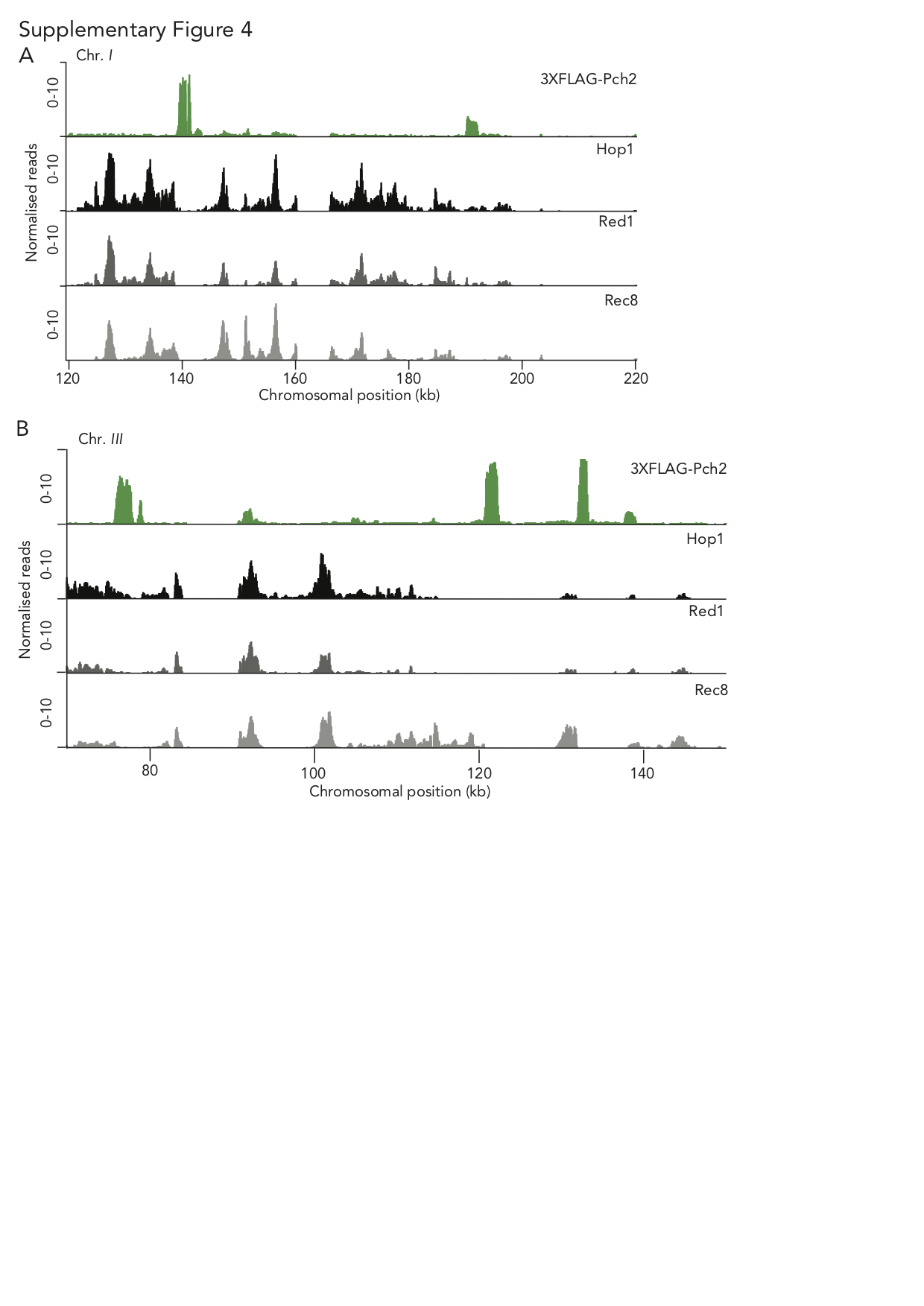

Supplement: S4 Fig — A and B. Representative images of ChIP-seq binding patterns for 3XFLAG-Pch2, Hop1, Red1 and Rec8. Data for Hop1, Red1 and Rec8 are from [9]. Shown is the entire chromosome I (A) and a region of chromosome III (B) (chromosomal coordinates (kb) are indicated). (TIFF) [file pgen.1008905.s005.tiff]

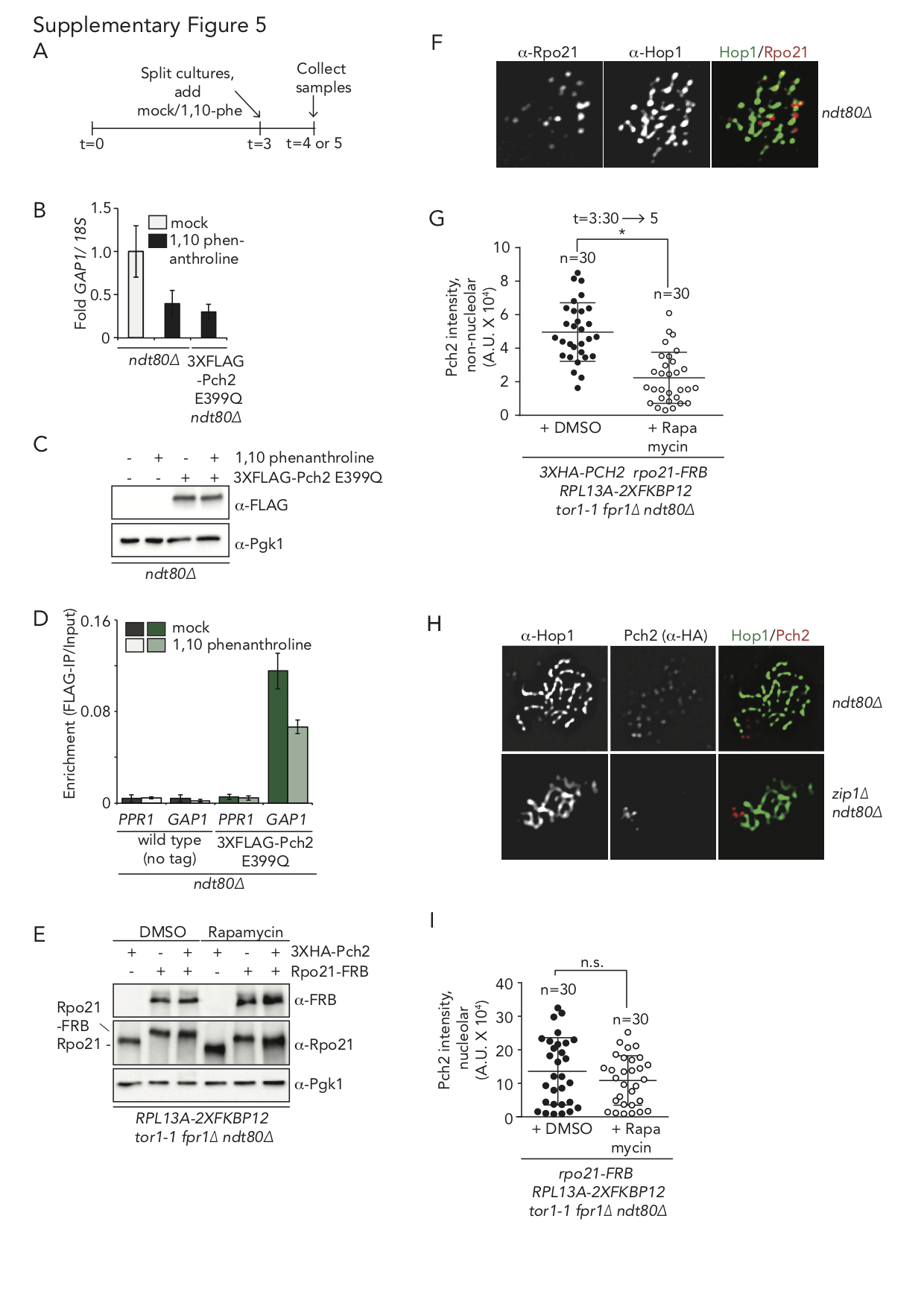

Supplement: S5 Fig — A. Schematic of 1,10- Phenanthroline treatment regimen as used for C-D B. mRNA quantification of GAP1 (primer pair: GV2597/GV2598)/18S (primer pair: GV2717/ GV2718) in cells (wild type or 3XFLAG-Pch2-E399Q) treated with mock (20% EtOH) or 1,10- Phenanthroline (final concentration is 2% EtOH and 100 μg/ml 1,10- Phenanthroline). C. Western blot analysis of 3XFLAG-Pch2 in cells (wild type or 3XFLAG-Pch2-E399Q) treated with mock or 1,10- Phenanthroline. D. ChIP-qPCR analysis of 3XFLAG-Pch2-E399Q at PPR1 (primer pair: GV2390/yGV2391), GAP1 (primer pair: GV2597/GV2598) in cells (wild type or 3XFLAG-Pch2-E399Q) treated with mock or 1,10- Phenanthroline. Error bars represent standard error of the mean of at least three biologically independent experiments performed in triplicate. E. Western blot analysis of Rpo21 (α-FRB or α-Rpo21) in wild type and rpo21-FRB anchor away strains, upon treatment with DMSO or rapamycin, treated as described in Fig 3C. F. Representative image of immunofluorescence of Hop1 and Rpo21 on meiotic chromosome spreads. G. Quantification of non-nucleolar Pch2 intensity per spread nucleus in cells treated with DMSO or rapamycin for 90 minutes, according to the scheme indicated in Fig 3C. H. Representative image of immunofluorescence of Pch2 on meiotic chromosome spreads in ndt80Δ and ndt80Δzip1Δ cells, as quantified in 3H. I. Quantification of nucleolar Pch2 intensity per spread nucleus in cells treated with DMSO or rapamycin. n.s. (non-significant) indicates p>0.05, Mann-Whitney U test. (TIFF) [file pgen.1008905.s006.tiff]

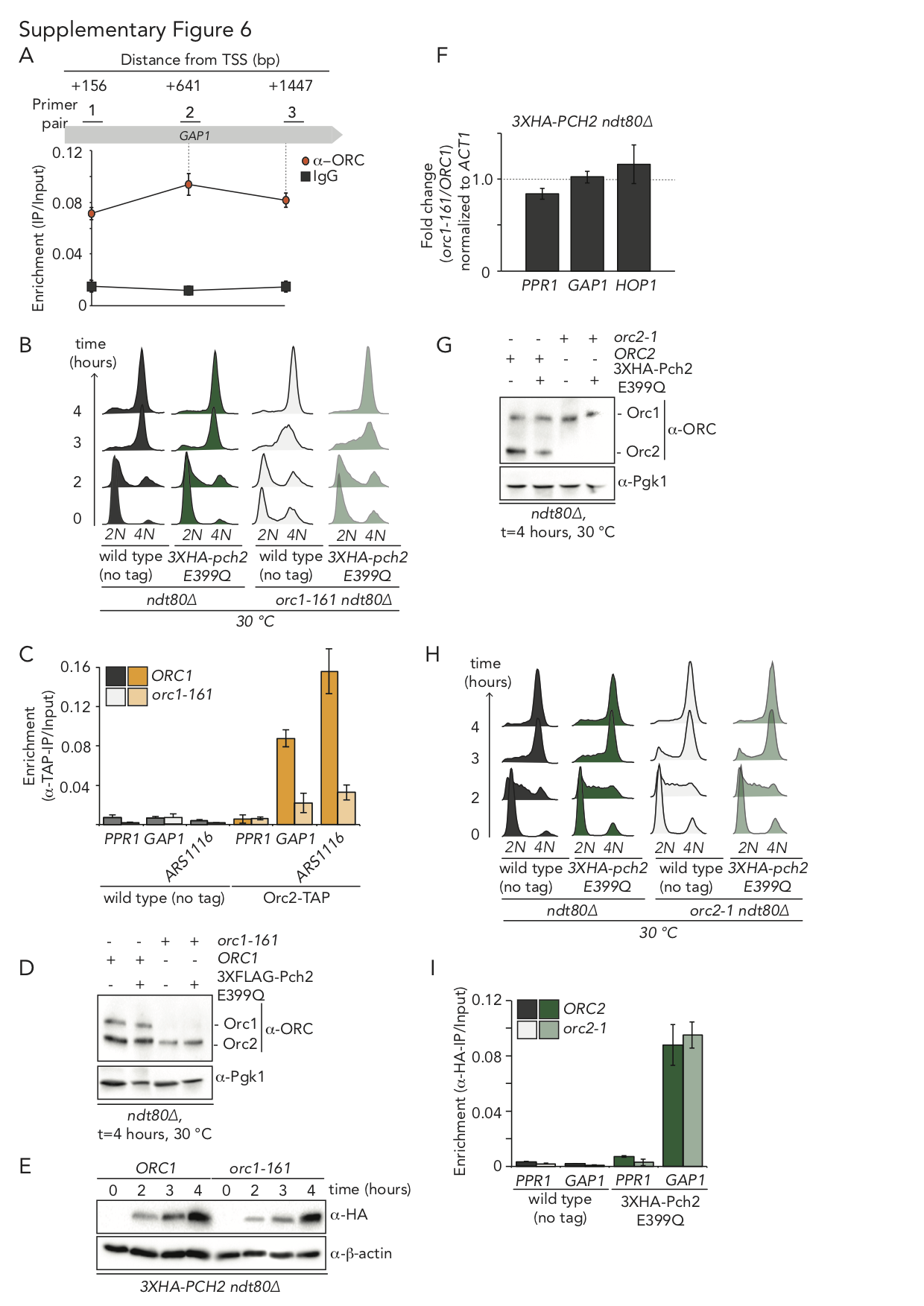

Supplement: S6 Fig — A. ChIP-qPCR analysis of ORC (α-ORC) along the GAP1 locus during meiotic G2/prophase (4 hours). Primers pair 1: GV2595/GV2596, 2: GV2597/GV2598, 3: GV2599/GV2600. Error bars represent standard error of the mean of at least three biologically independent experiments performed in triplicate. B. Flow cytometric analysis of ORC1 and orc1-161 cells (wild type, or expressing or 3XHA-Pch2-E399Q). Time (hours) after induction into the meiotic program is indicated. Experiment was performed at 30°C. C. ChIP-qPCR analysis of Orc2-TAP at PPR1 (primer pair: GV2390/GV2391), GAP1 (primer pair: GV2597/GV2598) and ARS1116 (primer pair: GV2577/GV2578) in ORC1 and orc1-161 cells. Experiment was performed at 30°C. Error bars represent standard error of the mean of at least three biologically independent experiments performed in triplicate. D. Western blot analysis of Orc1 and Orc2 (α-ORC) in ORC1 and orc1-161 cells. Experiment was performed at 30°C. E. Western blot analysis of 3XHA-Pch2 (α-HA) in ORC1 and orc1-161 cells. Hours after induction into the meiotic program indicated. Experiment was performed at 30°C. F. Gene expression of ORFs PPR1 (primer pair GV2390/GV2391), GAP1 (GV2597/GV2598), and HOP1 (primer pair GV2605/GV2606) relative to β-Actin (ACT1; primer pair GV2717/GV2718) in orc1-161 and ORC1 cells grown at 30°C. Relative gene expression of the reference strain (ORC1) was set to 1. G. Western blot analysis of Orc1 and Orc2 (α-ORC) in ORC2 and orc2-1 cells. Experiment was performed at 30°C. H. Flow cytometric analysis of ORC2 and orc2-1 cells (wild type, or expressing or 3XHA-Pch2-E399Q). Hours after induction into the meiotic program indicated. Experiment is performed at 30°C. I. ChIP-qPCR analysis of 3XHA-Pch2-E399Q at PPR1 (primer pair: GV2390/GV2391) and GAP1 (primer pair: GV2597/GV2598) in ORC2 and orc2-1 cells. Experiment was performed at 30°C. Error bars represent standard error of the mean of at least three biologically independent experiments performed in triplicate. [file pgen.1008905.s007.tiff]

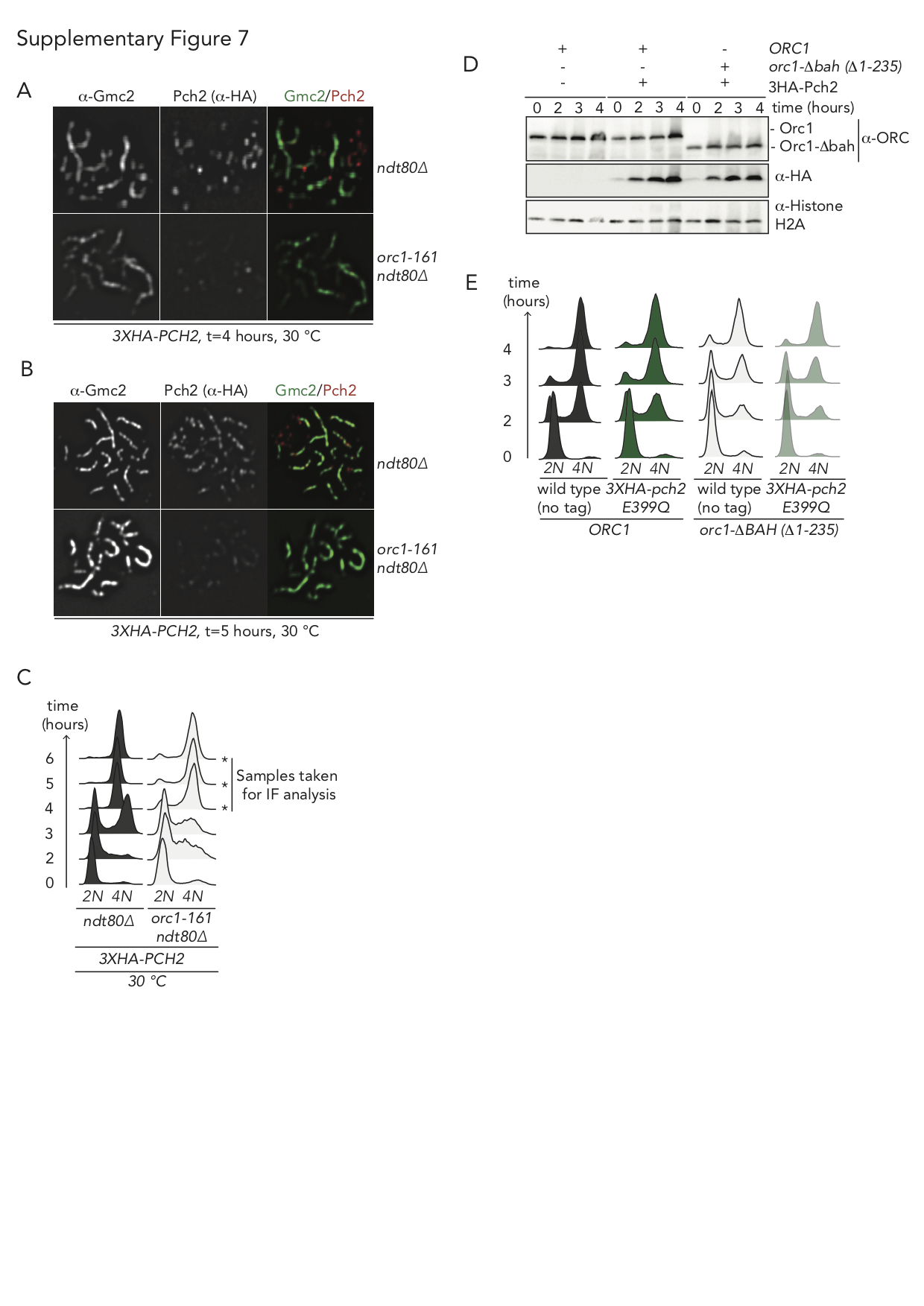

Supplement: S7 Fig — A and B. Representative images of immunofluorescence of meiotic chromosome spreads in 3XHA-Pch2 expressing ORC1 or orc1-161 cells collect at 4 hours (A) or 5 hours (B) after induction into the meiotic program. Experiments were performed at 30°C. For quantification see Fig 4G. C. Flow cytometric analysis of ORC1 and orc1-161 cells of cells that were analyzed in Fig 3F and 3G, and S7A and S7B Fig. Time (hours) after induction into the meiotic program is indicated. Experiment was performed at 30°C. D. Western blot analysis of 3XHA-Pch2 and Orc1 (α-HA and α-ORC) in ORC1 and orc1Δbah cells. Hours after induction into the meiotic program indicated. E. Flow cytometric analysis of ORC1 and orc1Δbah cells (wild type, or expressing 3XHA-Pch2-E399Q). Hours after induction into the meiotic program indicated. (TIFF) [file pgen.1008905.s008.tiff]

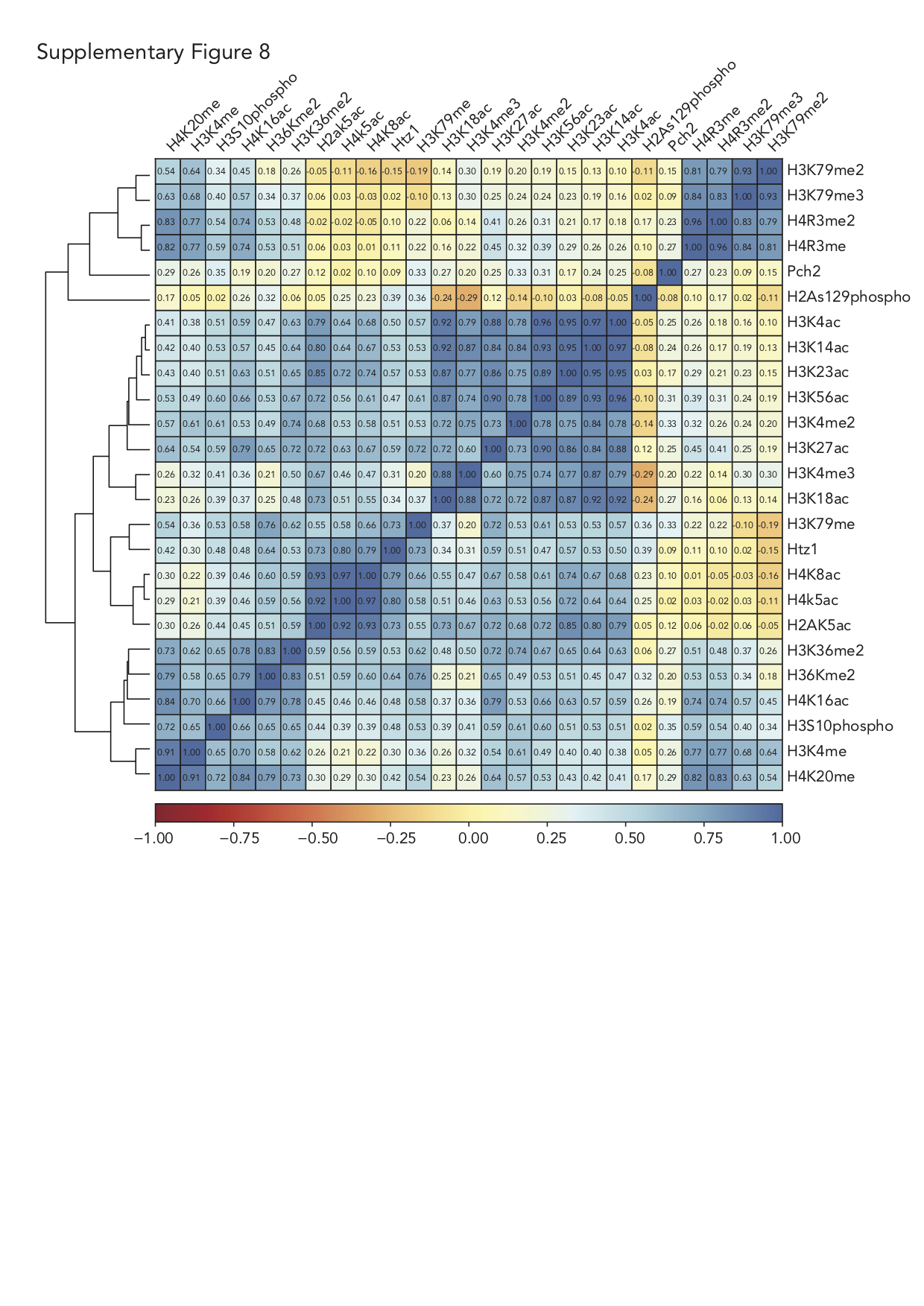

Supplement: S8 Fig — Hierarchically clustered heatmap based on correlation coefficients using from 3XFLAG-Pch2-wild type ChIP-seq datasets as inputs. Data for histone modifications are from [56]. Spearman correlation values are indicated. (TIFF) [file pgen.1008905.s009.tiff]

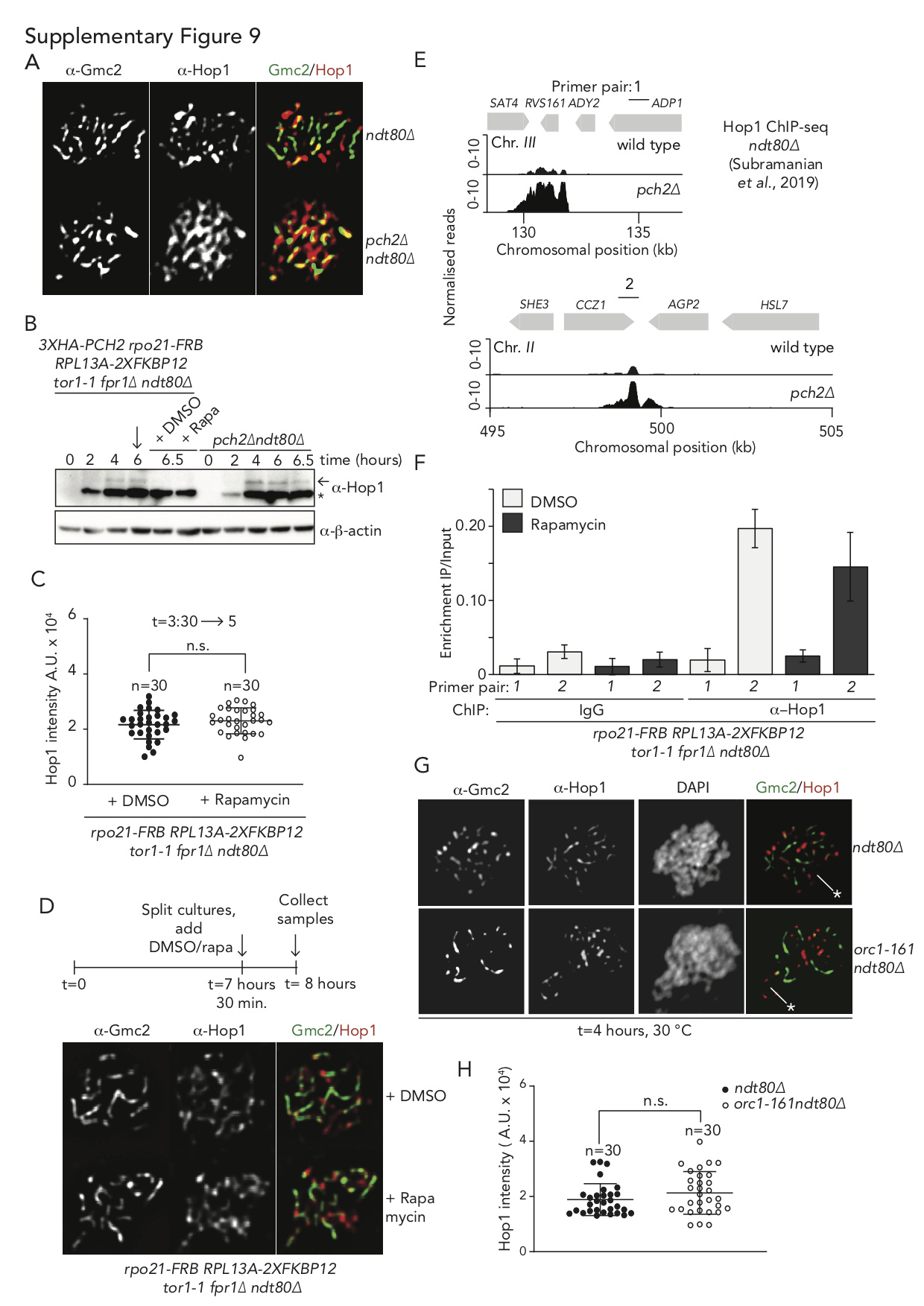

Supplement: S9 Fig — A. Representative image of immunofluorescence of Hop1 and Gmc2 on meiotic chromosome spreads in pch2Δndt80Δ and ndt80Δ cells. B. Western blot analysis of Hop1 and Pch2 (α-HA) in rpo21-FRB anchor away ndt80Δ and pch2Δndt80Δ cells progressing synchronously through meiotic prophase, treated with DMSO or rapamycin, as indicated above the western blot image. Arrow indicates phosphorylated Hop1, * indicates non-phosphorylated Hop1. C. Quantification of total Hop1 intensity per spread nucleus after treatment as indicated. n.s. (non-significant) indicates p>0.05, Mann-Whitney U test. D. Schematic of treatment regimen used for anchor away experiment for which quantification is shown in Fig 5B. Representative immunofluorescence of meiotic chromosome spreads in the 3XHA-Pch2 expressing rpo21-FRB anchor away cells, treated with DMSO or rapamycin as indicated. Chromosome synapsis was assessed by α-Gmc2 staining. E. Representative images of Hop1 ChIP-seq binding patterns in wild type and pch2Δ from [44]. Chromosome coordinates and primer pairs are indicated. F. ChIP-qPCR analysis of Hop1 in rpo21-FRB anchor away strains, upon treatment with DMSO or rapamycin, treated as described in Fig 2C. Primers are described in [44]. Error bars represent standard error of the mean of at least three biologically independent experiments performed in triplicate. G. Representative images of immunofluorescence of Hop1 on meiotic chromosome spreads in ORC1 or orc1-161 cells collect at 4 hours after induction into the meiotic program. Experiments were performed at 30°C. Chromosome synapsis was assessed by α-Gmc2 staining. *Indicates nucleolar region. H. Quantification of total Hop1 intensity per spread nucleus (as shown in S4G Fig) after treatment as indicated. n.s. (non-significant) indicates p>0.05, Mann-Whitney U test. (TIFF) [file pgen.1008905.s010.tiff]

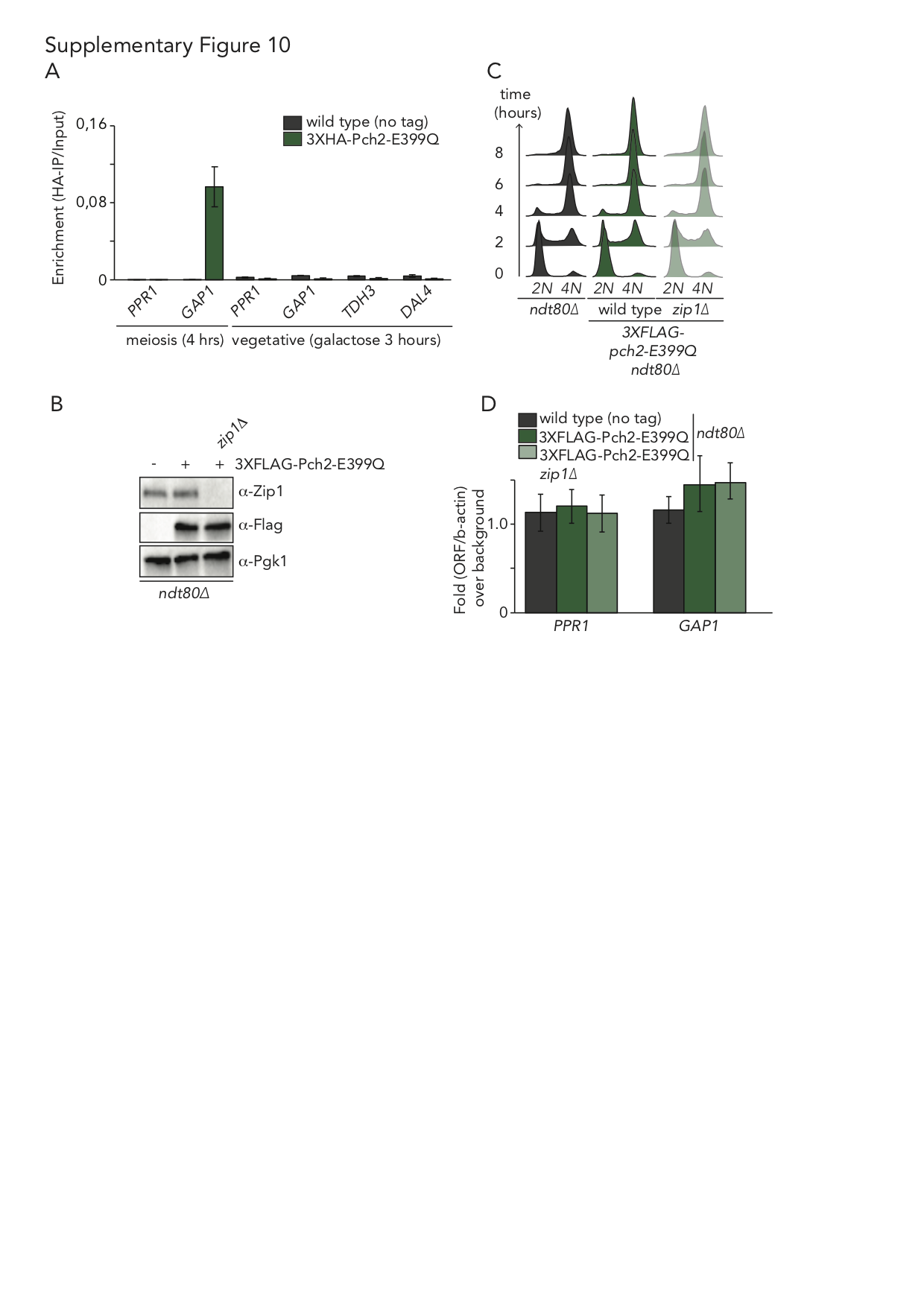

Supplement: S10 Fig — A. ChIP-qPCR analysis of 3XHA-Pch2-E399Q at PPR1 (primer pair: GV2390/GV2391), GAP1 (primer pair: GV2597/GV2598) during meiosis, and of PPR1 (primer pair: GV2390/GV2391), GAP1 (primer pair: GV2597/GV2598) and TDH3 (primer pair: GV2591/GV2592) and DAL4 primer pair: GV2601/GV2602) during mitosis. Time (hours) is indicated. Error bars represent standard error of the mean of at least three biologically independent experiments performed in triplicate. B. Western blot analysis of 3XFLAG-Pch2 and Zip1 (α-FLAG and α-Zip1) in wild type and zip1Δ cells. Time (hours) after induction into the meiotic program is indicated. C. Flow cytometric analysis of wild type and zip1Δ cells (wild type, or expressing 3XFLAG-Pch2-E399Q). Time (hours) after induction into the meiotic program is indicated. D. mRNA quantification of GAP1(primer pair: GV2597/GV2598) (primer pair: GV2717/GV2718) or PPR1 (primer pair: GV2390/GV2391)/β-Actin (ACT1; primer pair: GV2747/GV2748) in wild type or zip1Δ cells during meiotic G2/prophase (4 hours). (TIFF) [file pgen.1008905.s011.tiff]

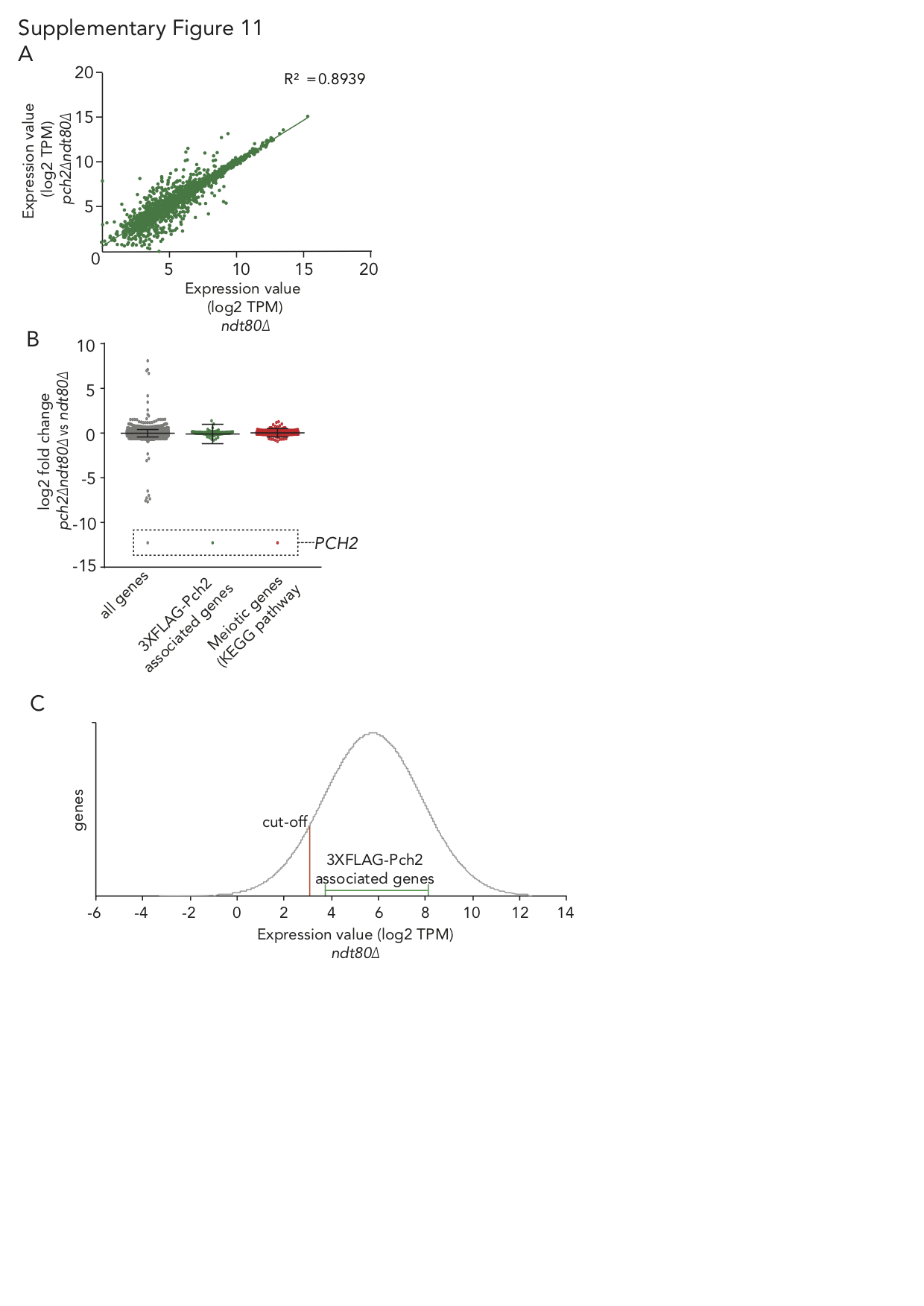

Supplement: S11 Fig — A. Pearson’s correlation analysis between expression levels (log2 TPM) of ndt80Δ and pch2Δndt80Δ. B. RNA seq-differential expression values from pch2Δndt80Δ relative to ndt80Δ, for all genes, wild type Pch2-binding and KEGG pathway (meiosis) genes. C. Histogram depicting the Log2 normal distribution of averaged TPM values from ndt80 strains RNA-seq. The defined expression cut-off (red line, see methods) and the range of TPM of 3X-FLAG-Pch2-wild type binding genes (green line) are indicated. (TIFF) [file pgen.1008905.s012.tiff]
