# Supplementary material for: Active transcription and Orc1 drive chromatin association of the AAA+ ATPase Pch2 during meiotic G2/prophase
Source: PLoS Genet. 2020 Jun 22;16(6):e1008905. doi: 10.1371/journal.pgen.1008905 (PMC7332104; doi:10.1371/journal.pgen.1008905)
Supplement: Attachment — Submitted filename: 2020 Review of revision of ORC1 paper.pdf [file pgen.1008905.s019.pdf]

2020 Review of revision of ORC1 paper

The authors have made some modest improvements to the presentation of their work. In particular, they have replaced the use of Peak shape score with a more simple presentation of ChIP-seq reads per million. Nevertheless, a number of outstanding queries arise regarding the calculation and presentation of data using this new metric (specific comments below), plus a number of points that were raised during the first round of revision but have not been answered or addressed.

General comment:

1. Despite the author's response, I still find the text to have unnecessarily large blocks of text, hampering readability and therefore critical understanding. For example, the entire introduction (2 pages, has only 4 paragraphs). Within the results, there is not a single paragraph break on page 5 nor on page 8, and only one on each of pages 10-13. These sections cover and discuss a lot of complex data, frequently pointing between main and supplementary figures. Giving the text (and the reader!) more space to breathe and take stock of what is being presented in each section of results would really help.

Specific comments:

1. Line 139-141. We respectfully request (again) that the correlation between repeat datasets are presented in the manuscript. The methods (Line 624) indicate that a program: multiBAMsummary (v 1.2) was used. Please describe what quantitative correlation this software measures, what bin size was used (and justify its use), and then state the pairwise results between the replicate datasets. I note that the default bin size is 10 kb (https://deeptools.readthedocs.io/en/develop/content/tools/multiBamSummary.html), which seems inappropriate for these data.

Although it may be standard practice to plot/compute the correlation between binned data in this way, if much of the read data are noise (i.e. relatively uniform), which is quite likely in ChIP-seq data, it may make more sense to compute the correlation only between the signals present within the called peaks, or to threshold the data first so that all the low occupancy (noisy) bins are ignored in the correlation.

Moreover, were the same number of peaks called in every dataset? Were they in the same position?

Finally, as previously suggested, presenting replicate datasets as overlays (to demonstrate whether or not each sample has a relatively similar profile pattern) would be a visual way help convince the reader that what is being presented is both reproducible and meaningful.

2. I appreciate the replacement of the Peak shape score metric with one that is more a direct measure of occupancy at each location. However, this metric is still not well described. What are the units for example? I assume it is reads/million mapped? But is this per bp, or per kb, or something else?

Further: how is this metric affected by the “normalisation against input”. How was this done? Were data binned and then divided? At what bin-size? Is the final value therefore a ratio? If so, should the data be plotted on a log scale of enrichment similar to other plots in the paper? (e.g. Fig 1E). This information needs to be added to the methods and summarised in the legend/figure.

Moreover, why have the “input” controls profiles been removed from the revision? They are valuable information because they enable the reader to appreciate the inherent “peaky-ness” (or not) of the input relative to the actual quantified data.

One of our question has not been answered, and still stands:

“What if most of the signal is dispersed?…Do peaks represent the majority, or the minority, of the signal reported?”

3. The fact that 98% of called peaks overlap RNAPolI genes sounds convincing. However, (as above): A) what fraction of the total ChIP-seq reads did this represent? B) What is the expected (random) overlap given the average width of the called peaks and the fraction of the genome that is an RNA Pol II gene?

More significantly, given that the ChIP-seq signal can arise via an indirect crosslink between the antigen and a histone (within the genic regions) and the DNA, it might be expected that signal would preferentially arise within gene bodies where histones have greater occupancy levels. Perhaps comparing the distribution of Pch2 to that of other factors (that don’t enrich in gene bodies) would help make the distinction here.

In fact – an overlay on Fig 1D of Hop1/Red1 etc would really be useful in this regard because it is the only figure that shows the relationship between the ChIP-seq profiles and gene structure, which itself is a major finding of the study.

9. Supplying the raw data is important, but supplying a large table is not very helpful for the average reader. Moreover, my original query remains answered: “A correlation of the shared set of peaks would be informative to determine if, on average, the same peaks are stronger in the mutant (or variable?), or whether the global increase reported is because they represent different peaks detected in the mutant. “

Line 166-169. It is (still) not clear where the data that underpin this statement is presented in the manuscript. This is an important point in the text. Why are there no figures to support this statement?

The addition of Fig 1F (And associated description in the text is welcomed). I would only ask for Fig 1F to be plotted in a square form such that the total spread of the dats in the X and Y directions are similar. As presented this is far from the case, with no apparent justification.

The authors may also wish to consider that these raw data actually reveal two clusters of data (zero/noise expression and non zero/real expression)—where neither display a quantitative correlation with Pch2 occupancy as measured by RNA-seq and ChIP-seq:

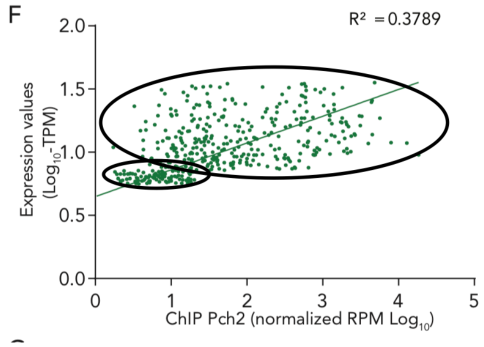

I leave it for the authors to decide whether or not to make any changes to their interpretations.

16. Do the authors mean to refer to Fig 3I in their response? If so, where do the data indicate that the loss in zip1 ndt80 is greater than the loss in the anchor-away system? This difference does not appear to have been tested statistically. Interpretation here is unclear: do the authors consider that this difference is important?

18. It would make more sense to describe the results as demonstrating that not only was there no enrichment of Pch2 around ARSs, that ARSs actually displayed local depletion for Pch2 binding (since this is the most obvious feature of the plot presented in Fig 4B).

19. Line 973 and Fig 2B. Something weird has gone on with the labelling here. I think the strains on the X-axis (top) are in the reverse order. Please correct.
Line 973. Should this say Pearson or Spearman…? Please describe how these correlations were calculated. Were the data binned before analysis? At what resolution? Were data thresholded first?

22. The rewording (lines 378-381), is still very hard to follow. I suggest to replace: “based on published Hop1 ChIP-seq datasets in wild type and pch2Δ cells [44]” with “around a known Hop1-binding site [44]…”.
