# Supplementary material for: Active transcription and Orc1 drive chromatin association of the AAA+ ATPase Pch2 during meiotic G2/prophase
Source: PLoS Genet. 2020 Jun 22;16(6):e1008905. doi: 10.1371/journal.pgen.1008905 (PMC7332104; doi:10.1371/journal.pgen.1008905)
Supplement: Attachment — Submitted filename: Silva_point-to-pointreply.docx [file pgen.1008905.s018.docx]

Dear Editor,

We thank the reviewers for their constructive comments on our manuscript. We have attempted to address concerns and questions, and outline detailed responses in our point-to-point reply below.

*Comments from Associate Editor:*

*1. A major concern that I (ML) had, and that reviewers expressed, involves the use of "peak shape score" as a proxy for occupancy in the analysis of ChIP-seq data. As far as I can tell (and the reference describing this metric is pretty opaque on the issue), this is a metric that can be independent of actual occupancy levels--for example, a sharp peak in a region of low background occupancy could have a higher score than a broad peak of higher occupancy levels in a region of high background occupancy. My suspicion is that differences in actual occupancy profiles in the ChIP-seq experiments (i.e. ChIP/input) are much smaller, and it is of course this value, not one reflecting peakiness, that is of actual biological relevance. Given this concern, I think that it is important to provide corresponding ChIP-seq profiles that report ChIP/input ratios for the regions examined (e.g. Figure 1C, D, Fig 4A, Fig S5, Fig S6F) and to provide ChIP-qPCR values for primer pairs at peak loci (i.e. GAP1, HOP1, etc) not only within the "peak" area but also outside them. I think that it is likely that the reduction in Pch2 occupancy seen under conditions of Pol2 inhibition/depletion and of ORC mutants are going to turn out to be regional rather than gene body-specific, but of course that remains to be seen. I believe that this concern is also what underlies reviewers' requests for additional ChIP-seq data. It must be emphasized that this is a critical concern; until true occupancy data are in hand, it will not be possible to evaluate the biological significance of the experiments.*

We apologize if our representation of the ChIP-seq datasets was unclear. To clarify: the way the data was presented (as peak-shape scores) was normalized against input, but nonetheless, we have now re-analyzed our data sets as suggested using conventional analyses that report on true occupancy. We are now using ‘number of reads/millions of total mapped reads’ normalized against input. We present revised representative images/presentations that show or use normalized reads score in our revised manuscript (*i.e.* Figures 1C-E, 2A, 4B, and Supplementary Figures 1D-F, 2A and B, 3A and B, 7, 8E). We also have performed all the downstream analysis of our datasets based on this new mapping, and again all presentations now are using those analyses. Most importantly, the conclusions as initially presented do not change after this new analysis: we equally observe a specific association of Pch2 with gene-bodies associated with active transcription. Further corroborating these conclusions, we now also, as requested, have expanded our ChIP-qPCR analysis, to include regions “outside” of the gene-bodies (i*.e.* directly upstream and downstream of a gene body). This analysis is presented for *GAP1*, *TDH3* (two Pch2-bound genes) and *PPR1* (a negative control) in Figure 1F. While we completely agree that performing additional ChIP-seq analysis of Pch2 in different mutant background (*e.g*. *orc1-161*, RNAPII-inhibition) would be extremely interesting, we have unfortunately not been able to perform such ChIP-seq datasets, due to a combination of time and technical limitations. We do believe that our extensive follow-up analysis based on ChIP-qPCR and cytological analyses provides solid evidence supporting our model in which Pch2 is specifically recruited to defined regions of transcriptional activity.

*2. A second concern involves the experiments with orc mutants. In general, the defect in Pch2 occupancy seems to parallel defects in replication; orc1-161 and orc1-∆BAH show delayed replication, while orc2-1does not. It seems quite possible that, at the 4h time point when ChIP is done, some aspect of meiotic chromosome structure is incompletely established in mutants where replication is compromised, and this, rather than a direct Orc1 interaction, is responsible for the observed effects. Unless the Pch2-Orc1 interaction can be disrupted without affecting replication dynamics, this will remain an open question, and the conclusions regarding the contribution of Orc to Pch2 recruitment will need to be substantially revised.*

We share the concern regarding a connection between DNA replication delays and Pch2 recruitment defects as seen in *orc1-161* and *orc1-Δbah* cells. We have now performed additional immunofluorescence (IF) analysis of Pch2-association with meiotic chromosome spreads in wild type and *orc1-161* mutants at different time points during meiotic progression (presented in Figure 4F and G, and Supplementary Figure 6A and B). Under the tested conditions, we consistently observed a delay of ~1 hour in completing pre-meiotic DNA replication in *orc1-161* mutant cells, as judged by flow cytometry (see for example Supplementary Figures 5B and G, 6C and E). When taking this in consideration, and assuming that defects in DNA replication (and associated delays in chromosome metabolism) are the underlying cause of reduced Pch2 localization under such conditions, one would expect that at later time points in *orc1-161* cells Pch2 localization should be restored to levels comparable to those seen in wild type cells. We, however, do not observe such a behaviour: in *orc1-161* cells we consistently, even at t=5 or t=6 hours, observe significantly reduced levels of Pch2 associated with meiotic chromosomes as compared to wild type cells, also when comparing wild type cells (at t=4 hours) to *orc-161* cells (t=5 or 6 hours) (See Figure 4G). It is important to note that for this analysis, *i)* we used *ndt80Δ* to prevent exit from G2/prophase at later time points, *ii)* we selected cells based on similar synaptonemal complex (SC) assembly status as judged by Gmc2 staining (for example of this, see Figure 3F, and Supplementary Figure 6A and B).

In addition, we would like to emphasize that in a parallel study from our lab [1] we have found, using purified components, that Pch2 forms a complex with ORC *in vitro*, demonstrating that these factors in fact directly interact. In the same study, we also provide evidence that inhibiting DNA replication (using a *CDC6* depletion allele; *cdc6-mn*) does not trigger the same (rDNA-associated) phenotype as what is observed in *orc1-161* cells. In addition, the *in vivo* and *in vitro* interaction between Pch2 and ORC occurs independently of Cdc6, providing evidence for a direct connection between ORC and Pch2, in a manner independent of the canonical role for ORC in DNA replication.

We agree that employing a point mutant that can uncouple Pch2-Orc1 binding from DNA replication dynamics would be ideal. However, because of the reasons listed above, we are confident that the effect of *orc1* mutants on Pch2 chromosomal association is a direct consequence of the association between Pch2 and ORC. Nonetheless, as suggested, we have reworded our statements in manuscript to reflect the fact that we cannot completely rule out a contribution of DNA replication efficiency on our observations.

*3. (comments 1 and 2 are in letter body, above). PLOS has a very strict data availability policy (please see letter body, above and* [*journals.plos.org/plosgenetics/s/data-availability*](http://journals.plos.org/plosgenetics/s/data-availability)*). In particular, the term "data not shown" is verboten; either show data or revise the text so that the statement is not necessary.*

*a. Relevant lines in the manuscript are 157, 160-4, 252, 254, 329 and 392; for line 153, I suggest that you simply say that the current paper describes Pch2 occupancy patterns in non-rDNA sequence, and that binding patterns in rDNA will be described elsewhere.*

We have now included a brief section in the result section where we discuss the general binding of Pch2 within the rDNA locus, and the deposited ChIP-seq datasets include rDNA sequences as well. However, while we obviously agree that understanding rDNA-recruitment of Pch2 warrants further study, we feel that including additional experiments to confirm and investigate these binding patterns further is beyond the scope of this manuscript.

*b. The ChIP-seq data need to be made available to reviewers, so please give the password in manuscript on line 521--reviewers do not have access to the cover letter.*

We apologize for this omission. The password is now included in the main text.

*c. For all figure panels with graphs, please provide the data underlying each graph. This can most easily be by including an Excel workbook with a separate worksheet for each panel. For example, for Figure 1F, please include all values for each primer pair in both tagged and untagged strains. (This need not be done with very data-dense panels such as 1C, D or E).*

We have now included a supplementary data file where we report on all the data underlying ChIP-qPCR graphs as they appear in the manuscript.

*4. Figure legends--I assume that by “standard error” you mean standard error of the mean; please clarify, and also please clarify what “at least three independent experiments performed in triplicate” means—and how SEM was calculated. Were there three sets of three values, or just three values?*

Indeed, standard error of the mean was meant here. Data is from three independent, biological replicates. This is now explicitly mentioned in the manuscript, and we also include a description how SEM was calculated.

*5. Fig S1C—correlation seems to be driven entirely by genes with no expression. Once these are eliminated, the is probably not correlation at all.*

We have re-analyzed our data using a new RNA-seq dataset (also presented in our manuscript) and have plotted the same graph. We now obtain a low positive correlation between expression values and Pch2 binding scores (R^2^ = 0,3789), suggesting at least a weak relationship between transcriptional activity and Pch2 association. However, as also indicated in Supplementary Figure 1E, Pch2 binding sites show a broad distribution for transcriptional strength, suggesting the presence of additional factors that influence binding patterns.

*6. line 67: Red1 shares no homology to SYCP2/3; better to make clear that the proteins are functional analogs but do not share significant homology.*

We have now changed this statement to include that these factors are functional and structural homologs, and we include a reference to support this claim [2].

*7. line 137: Capitalize Materials and Methods; please use 10(superscript)-15 rather than 1e-15*

This has been changed accordingly.

*8. Figure 1F—given the apparent absence of Pch2 from sequences up- and down-stream of GAP1, it would seem a good idea to include qPCR primers for these regions, as well; this would be a good control for the Pol2 ChIP as well (see AE comment 2, above).*

We have now included qPCR analysis of flanking regions for Pch2 ChIP at *GAP1*, *TDH3* (two Pch2-bound genes) and *PPR1* (a negative control) in Figure 1F. See also response to AE comment 2.

*9. Figure 1G—please quantify FLAG/Pgk1 ratios; it appears that Pch2 243-564 is present at substantially lower levels. Is Pch2 243-564 localized to the nucleus?*

We have now included the quantification of three different western blot analyses in the Supplementary Table 4. Indeed, Pch2 243-564 is present at lower levels. We have not tested whether this mutant is localised in the nucleus. Based on our *in vitro* analysis of Pch2 and ORC [1], Pch2 lacking the NTD (*i.e.* Pch2 243-564) appears to form poorly structured hexamers, suggesting that the NTD actively contributes to hexamerization. In addition, recent work from the San-Segundo lab demonstrated that the NTD of Pch2 contains a NLS; deleting the entire NTD is thus expected to impairs the proper nuclear localization of Pch2 [3]. We thus suspect that Pch2 243-564 is not efficiently incorporated into the nucleus. We have mentioned these observations in the revised manuscript.

*10. Other western blots—please quantify and present ratios for the following; Figure 1B, 2G, 4F (including upshifted Hop1), 5B, S4B, S4E, S4F, S4I. For Figure S7C, it will be important to have the Zip1 and zip1∆ samples on the same blot, to determine whether or not Pch2-E399Q levels are similar in both strains. Ratios can be reported under each lane, or, if it makes figures too crowded, in a supplementary table.*

We have now included the quantification of selected western blots in a Supplementary Table 4. We have also replaced the western blot in Supplementary Figure 9B so that *ZIP1* and *zip1∆* samples are on the same blot.

*11. Figure S1B—why is RNA-seq under High, Medium and Low, while mRNA levels is under all 4?*

We apologize for this, and have clarified the figure.

*12. Figure S3A—was the phenanthroline treatment here as shown in panel B?*

Yes, the treatment was as indicated in panel B, and we now mention this in the manuscript*.* In order to clarify the outline of this Supplementary Figure we have moved panel B to A and *vice versa*.

*13. Figure S4F—at what time in meiosis were these protein samples taken?*

The samples were taken at t=4 hours, and this is now mentioned in the Figure legends.

*14. Figure S6F—please provide Y axis labels and values. I could find no such images in Subramanian et al, and am assuming that you re-analyzed their data. Please provide full information for this analysis, including the datasets analyzed, method used, etc.*

For this figure, the peaks were extracted from the GEO datafiles associated with the referred paper [4] and plotted directly as indicated. We now describe this in the Materials and Methods.

*15. Please proofread references and correct title capitalization, italicization of genes and species, journal abbreviations, etc.*

This has been changed accordingly.

*16. Please carefully read the instructions to authors (*[*https://journals.plos.org/plosgenetics/s/submission-guidelines*](https://journals.plos.org/plosgenetics/s/submission-guidelines)*) and follow formatting instructions (helpful examples can be downloaded) in crafting the revision. 

Reviewer's Responses to Questions


Reviewer #1: Notes on Pch2 PLoS Genetics Manuscript

In this manuscript, Cardoso da Silva et al. examine the meiotic chromosome localization of Pch2, a highly conserved and important regulator of meiotic recombination. The key experiment here - Pch2 ChIP-Seq - is of high interest to the field and one that is potentially very informative about Pch2's regulation and functions. It has the potential to help us understand Pch2's seemingly diverse roles: in controlling the localization of the recombination regulator Hop1 on chromosomes, in suppressing recombination in the repetitive rDNA, and in its mysterious checkpoint-like function in mutants with compromised inter-homolog synapsis.

While the topic is of high relevance and the data interesting, I can't recommend publication without significant revisions to address a number of major and minor questions/concerns, which are listed in detail below. In summary, while the authors have identified a set of Pch2-localizing sites on chromosomes in meiotic prophase, they do not adequately explain how Pch2 gets to these sites (yes, it's transcription and Orc1-dependent, but why?), nor do they reveal any biological role for these localization sites. That is, the sites identified do not seem relevant to Pch2's most well-recognized function, in Hop1 remodeling/removal; this may be a consequence of the depletions etc. being done too late in meiotic prophase to see a difference. The authors also inexplicably fail to present some seemingly very relevant data and analyses, noting them as "unpublished observations": this includes data on Pch2's localization to rDNA, which would arguably be one of the most interesting features of their ChIP-Seq dataset, and an analysis of Pch2 ChIP-Seq compared to various histone modifications. I am also somewhat concerned about the peak-calling algorithm, and features of some figures that suggest that the observed Pch2 localization is very weak.

Finally, I hate to suggest excessive extra work in a review, preferring instead to review the material as presented. Nonetheless I can't help but note that the manuscript would be much stronger if ChIP-Seq had been performed instead of ChIP-qPCR for many comparisons. This would allow genome-wide correlations of, e.g., Pch2 and Orc1 localization in meiotic prophase, Pch2 localization in mitotic cells, etc. etc. On a similar note, because meiotic prophase stage is so very important in understanding Pch2 function, a time-course for Pch2 localization (at selected sites by ChIP-qPCR or better, by ChIP-Seq) would be extremely informative.

Major comments/concerns:

1. Page 6 - The authors do most of their assays four hours after induction of meiosis. What stage of meiotic prophase are these cells in? I can imagine that catching Pch2 in its (presumably) most physiologically-relevant state - during synapsis, when it is localizing to chromosomes and remodeling/removing Hop1 from the axis - might be difficult to accomplish. If the cells are pre-zygotene or already in pachytene by this analysis, what is the relevance of the localization the authors are mapping?*

We provide an analysis of progression through meiotic G2/prophase, based on the progressive assembly of the synaptomenal complex (SC; as measured by Gmc2 staining, an SC component, Supplementary Figure 1C). This analysis shows that at 4 hours post-induction (the time of ChIP-seq analysis), cells are in the process of synapsing their chromosomes (*i.e.* we observe a majority of cells that exhibit either fully assembled SCs and/or partially assembled SCs) (note that this strain is *ndt80Δ,* to prevent meiotic progression past pachytene). During this time of meiotic G2/prophase, several processes, such as DNA breakage, DNA recombination, checkpoint signalling, and chromosome remodelling are in progress. Pch2 is known to contribute to all these processes, and its chromosomal localization should be prevalent under these conditions. We thus are convinced that analyzing the chromosomal localization of Pch2 during this time of meiotic G2/prophase is likely relevant to the functions of Pch2 in meiosis.

*2. Page 6 - Please describe "peak shape score" in more detail. From my quick literature* search, this seems to be a score generated by a robust "learning" peak-calling method, but it bears some explanation in the text. For example, what is a "good" peak shape score?

Please refer to point 2 of AEs comments. We have now re-analyzed the dataset using other methods (and thus do not use ‘peak shape scores’ anymore), and explain the results there.

*3. Page 6 - The authors note (several times) the fact that they observed Pch2 localization to the rDNA by ChIP, yet choose not to describe it in this manuscript. I can't see why it's excluded; it seems very relevant to our understanding of how Pch2 works.*

We have now included a brief section in the result section where we discuss the general binding of Pch2 within the rDNA locus, and the deposited ChIP-seq datasets include rDNA sequences as well. In brief, we found that Pch2 is associated with RNAPI-dependent transcripts (i.e. 25S), but not with RNAPIII transcription (i.e. it is not found on the 5S region). In addition, we do not find specific enrichment of Pch2 within the intergenic regions (*NTS1* and *NTS2*). We have mentioned these observations in our revised manuscript. However, while we obviously agree that understanding rDNA-recruitment of Pch2 is very interesting and warrants further study, we feel that including additional experiments to confirm and elaborate on these binding patterns is currently beyond the scope of this manuscript.

*4. Figure 1E does not make sense to me. If the authors are averaging the ChIP-Seq signal over ~500 peaks, why is the shape of this graph so jagged, instead of smooth? Also, why is the signal seemingly so low? It seems from the graph that the baseline level is 0.8 ChIP-Seq/Input (log2 ratio), while the peaks are only 0.92? Can this be right? The peaks are only 1.9-fold enriched over background binding, and the background is somehow 1.75-fold enriched over background? What's going on here? If these are the real ratios, is the peak-calling algorithm just enhancing noise? Figure 3B is also much more jagged than expected from an average of many peaks.*

We have now re-analyzed these plots (Figure 1E, Figure 4B and Supplementary Figure 2B) and indeed find a much stronger enrichment among gene bodies of Pch2 binding sites in the case of Figure 1E. We apologize for this erroneous analysis and thank the reviewer for pointing this out to us.

*5. Figure 2I, 3G, and (maybe) 4D) - It seems that counting the number of foci of Pch2 (Hop1 in Figure 4D) is not really the right way to quantify the data. There is obviously a dramatic reduction in overall intensity of chromosome-associated Pch2 in Rapamycin treatment and orc1-161 cells (assuming the panels are intensity-matched), but the number of foci changes much less than the overall intensity. Is there are way to quantify overall Pch2 intensity per nucleus, rather than number of foci?*

We have re-analyzed our data as suggested by the reviewer. Briefly, per cell, we define a surface based on Gcm2 staining (in order to exclude staining of Pch2 within the nucleus and thus to obtain data on “euchromatin”-only association), and use this to obtain the intensity normalized to background (in arbitrary units) of the Pch2 signal/cell. We use a minimum of 30 cells (from two independent experiments) per described conditions. This reanalysis indeed reveals a more significant effect of *orc1-161* and rpo21-FRB-driven depletion on Pch2 recruitment. We have now also included an analysis of Pch2 in a *zip1Δ* background to compare with our depletions/inactivation approaches (Figure 3F and Supplementary Figure 4H).

*6. Is ORC localized to origins of replication in meiotic prophase? If not, Pch2 localization there would not be expected.*

In Figure 4D and Supplementary Figure 5C, we have analyzed the localization of Orc1 and Orc2 to a defined origin (*ARS1116*). In addition, for another study from our lab (Villar-Fernandez *et al*. manuscript in revision) we have confirmed the association of Orc1-TAP with selected additional origins. Thus, although we have not performed a genome-wide analysis of ORC binding in meiosis, we are confident that ORC is associated with origins of replication during meiotic G2/prophase, which would also be expected based on the known behaviour of ORC during the budding yeast cell cycle.

*7. The authors cite a work by Shor et al. (ref. #45) that identified a large number of ORC-binding sites in highly-transcribed genes close to replication origins. Have the authors compared this set of genes to their own identified sites? TDH3, UTH1, and SSA1 are on the list of previously-identified "ORF-ORC sites" (not GAP1, however). The meiotic genes would not have been identified in that prior analysis, but it still may be a worthwhile comparison.*

We have compared the binding sites of ORC (ORF-ORC sites) from Shor *et al.* with our dataset. We now present a comparison of these sites in Supplementary Table 1. Briefly, out of our 437 Pch2 wild type binding sites, we found that 60 of those overlap with described ORF-ORC sites, out of a total of 163 sites found by Shor *et al.* [5]. Thus, although there are shared sites between the two datasets, the datasets also differ substantially. We now mention this in our manuscript. *8. On the subject of the above comment, it seems that Shor et al. showed that the "ORF-ORC" sites are all pretty close to origins. Have the authors looked at the proximity of their identified Pch2 peaks to origins, comparing that proximity to that of the typical gene to an origin?*

We agree with the reviewer that the finding by Shor *et al.* is potentially interesting. However, it remains unclear to us how this correlation between origin location and ORC-ORFs relates to the promotion of Pch2 binding to transcribed genes, especially since we do not find any association of Pch2 with origins of replication. We have chosen to not include any analysis pertaining to this observation by Shor *et al.* in our current manuscript.

*9. One more comment on the above, given the authors' experiments with Pch2 expression in mitosis seems to have shown no localization with GAP1, what about analyzing other sites? It seems that the authors should at minimum examine several other loci, or ideally perform ChIP-Seq again on these cells to compare all sites. It could be that GAP1 is atypical of sites (and HOP1 is not transcribed in mitosis - this is a fine negative control, but doesn't invalidate this point).*

We have included two other loci (*TDH3* and *DAL4*) in our ChIP-qPCR analysis in mitosis (shown in Supplementary Figure 9A), and like *GAP1*, Pch2 does not show association with these sites.

*10. Regarding the authors' result that deletion of the Orc1 BAH domain compromises Pch2 localization to gene bodies: Orc1's BAH domain is known to localize to H3K20 methyl marks, at least in other species. Can the authors comment on the possibility that the peaks they identify might be enriched in this mark, perhaps specifically in meiosis, for some reason?*

We assume the reviewer is referring to H4K20 dimethylation, a histone mark that contributes to the association of ORC to origins of replication in metazoans, via direct association of the BAH domain of Orc1 with H4K20-dimethylated nucleosomes [6]. However, the available data ([6]) shows that the BAH domain of yeast Orc1 is not able to associate with this methylated histone tail, suggesting that the BAH domain of budding yeast Orc1 does not specifically “read” this modification. In line with this, H4K20-di-methylation is current thought absent from budding yeast [7].

We have included a correlation matrix between Pch2 binding sites and a battery of other histone modifications for which there are good yeast ChIP-seq data sets available (from mitotic cells [8]). Interestingly, among other transcription-associated histone modifications, we find a positive correlation between Pch2 binding sites and H3K79-monomethylation. Dot1, the methyltransferase that methylates H3K79 is involved in proper Pch2 localization under certain conditions [9, 10], and the BAH domain of yeast Sir3 (which is similar to the BAH domain of Orc1) is sensitive to this modification with respect to nucleosome association [11]. This suggests that the observed binding pattern of Pch2 might be related to this particular histone modification, something that we now discuss in our revised manuscript. In addition to the correlation between Pch2 and H3K79-mono-methylation, we found that Pch2 correlates with several other marks, most notably H4K20-mono-methylation, H3K56-acetylation, H3K4-demethylation and H3S10-phosphorylation. A communality between these marks is their association with active RNAPII-transcription. The data comparing the histone modifications with Pch2 data sets is now included as Supplementary Figure 7, and we discuss these findings in the manuscript.

*11. Figure 4 - The experiment measuring Hop1 localization to chromosomes when disrupting Pch2 localization to gene bodies needs to be explained better. The interpretation of this data depends very strongly on what stage the cells are in during these measurements: If they are in leptotene/zygoetene, the authors can reasonably argue that the Hop1 they are measuring is the population of Hop1 that is promoting DSBs and COs prior to synapsis. If the cells are in pachytene during this analysis, then the authors are only looking at the "residual" Hop1 that remains on synapsed chromosomes, and is presumably not (or only weakly) promoting DSBs/COs. In the same vein, also matters a lot how long Pch2 localization has been compromised (i.e. if the Rapamycin was added after synapsis and the completion of Pch2's Hop1-removal task, then its depletion from chromosomes would not be expected to affect Hop1 levels.*

From earlier work [12], it is known that Hop1 chromosomal recruitment is highly dynamic during meiotic G2/prophase. Specifically, Subramanian *et al*. [12] showed that the depletion of Zip1 (using the anchor away approach) was sufficient to trigger visible effects on Hop1 within 60 minutes. We are therefore confident that under the used depletion times (that in our setup range from 30 to 90 minutes), we would be able to observe effects on Hop1 localization and phosphorylation. In addition, we perform our analysis “early” in meiotic G2/prophase (3:30-4 hours) and “late (*i.e*. 7:30-8:00).

*12. Related to the above point: Lines 333 and 334, the authors state that PNAPII depletion did not cause differences in the amount of phosphorlyated Hop1 (Figure 4E-F). But again this experiment was done entirely in pachytene, after the bulk of phosphorylated Hop1 had been removed/degraded/dephosphorylated. Depleting RNAPII during the meiotic prophase stage when Pch2 activity is most relevant for Hop1 (zygotene) would seem to be more informative.*

See also point 11, reviewer 1. In earlier work [12], it was shown that Hop1 chromosomal association is highly dynamic (and dependent on Pch2 activity), and could be observed in pachytene cells. We are therefore confident that during pachytene and/or zygotene, we would be able to observe effects on Hop1 localization (and phosphorylation).

*13. Discussion - the authors note that the Orc1 BAH domain may in fact associate with Dot1-mediated H3K79 methylated nucleosomes, and state that they detected a correlation between Pch2 ChIP-Seq and H3K79 methylation; but do not show this data. Why not? I think an important supplemental figure would be to show a correlation analysis of Pch2 ChIP-Seq peaks with every histone modification for which a dataset is available. Are any such datasets available from meiotic cells?*

Unfortunately, no meiotic datasets are available, but we have performed and now show the analysis with available mitotic datasets. See also point 10, reviewer 1.

*14. When discussing the loop-axis structure of meiotic chromosomes, the authors should cite two recent Hi-C papers by Muller...Koszul (Mol Syst Biol 14(7):e8293) and Schalbetter...Neale (Nature Communications 10(1):4795, October 2019) describing the yeast loop-axis structure in detail, and perhaps also the mammalian-cell Hi-C papers by Alavattam et al (NSMB 2019), Wang et al. (Mol Cell 2019), Patel et al. (NSMB 2019), and Covadonga Vara et al. (Cell Reports 2019)*

We have now included these references.

*Minor points:*

*1. Figure 1F - this would be much more useful if the authors added one or a couple probes outside the ChIP-Seq peak for GAP1.*

We have done this, see reply to point 1 of AE and Figure 1F. *2. Line 148: Reference Supplementary Fig. 1A.*

This has been changed accordingly.

*3. Line 185-188: Please double check with the figure reference.*

This has been changed accordingly.

*4. Line 234 - "PolI" should be "PolII"*

This has been changed accordingly.

*Reviewer #2: This work by Cardoso da Silva et al. reports the association of Pch2 with a subset of chromosomal regions containing genes that are actively transcribed by RNA polymerase II during meiosis. In fact, active transcription is required for Pch2 recruitment to those sites. Paralleling the previously known role of Orc1 (a subunit of ORC) in Pch2 localization to the rDNA, this study shows that Orc1, but not Orc2, is also required for Pch2 binding to selected gene bodies in euchromatin. However, unlike ORC, Pch2 is not detected at replication origins. In addition, Zip1 is also needed for Pch2 association to transcribed regions. However, impairment of Pch2 chromatin recruitment by depletion of the Rpo21 component of RNAPII does not result in any effect in Hop1 chromosomal distribution and abundance, suggesting that the chromosomal population of Pch2 described in this work is not involved in regulating Hop1 function.

Given the importance of Pch2 in the control of various aspects of meiotic chromosome metabolism, the findings reported in this manuscript are interesting in the meiosis field. The experiments performed in this work are very well executed, the results are clear and, for the most part, support the conclusions presented. The main weakness of this paper is that it does not provide any insight into the possible biological relevance for meiosis of the transcription-associated population of Pch2 reported here.

Major comments/concerns

1) Immunolocalization of Rpo21-FRB on chromosome spreads in the absence of rapamycin (+DMSO) shows a rather exclusive pattern with that of Zip1 in the single nucleus presented in Figure 2D. Curiously, this pattern is reminiscent of Hop1 localization, also displaying an alternative distribution with Zip1 on wild-type prophase I chromosomes (San-Segundo and Roeder, Cell 1999; Joshi et al., PLoS Genetics 2009). The authors could test whether Rpo21 foci, representing transcription sites, and Hop1 colocalize.*

We have now performed a co-localization analysis between Rpo21 and Hop1 (Supplementary Figure 4F). As can be seen from this analysis, Rpo21 forms distinct foci along meiotic chromosomes, but these foci appear (both qualitatively and quantitatively) different than Hop1: they are more focal (*i.e.* they don’t paint large chromosomal areas), and less abundant. We now mention this observation in the revised manuscript.

*2) Figures 2H, 2I (as well as Figures S6B, S6C) conclude that depletion of Rpo21 leads to fewer Pch2 chromosomal foci without affecting nucleolar Pch2. From the representative nuclei shown, it appears that, upon Rpo21 depletion, Pch2 foci are not only fewer, but also weaker. This raises the question of whether the remaining foci represent the same population of Pch2 that depends on transcription with lower amount of Pch2 loaded or, alternatively, that those Pch2 remaining foci correspond to an Rpo21-independent population and, therefore, may be involved in Hop1 chromosome exclusion. In other words, since by ChIP-seq only one chromosomal fraction of Pch2 is detected, which is transcription-dependent and not involved in Hop1 distribution, do the cytological assays detect only this same population? First, quantification of the Pch2 nucleolar signal relative to chromosomal Pch2 must be performed to definitely state that the nucleolar pool is unaffected. Second, colocalization between Hop1 and Pch2 before and after Rpo21 depletion must be assessed and quantified to determine if the population of Pch2 that remains after transcription inhibition shows a different chromosomal distribution relative to Hop1 or there is some degree of overlap. Also colocalization between Gmc2 and Pch2 (-/+ Rapamycin) should be quantified.*

We have re-analyzed our data as suggested by the reviewer. Briefly, on meiotic chromosome spreads, we define a surface based on Gcm2 staining (in order to exclude staining of Pch2 within the nucleus and thus to obtain data on “euchromatin”-only association), and use this to obtain the intensity normalized to background (in arbitrary units) of the Pch2 signal/cell. Conversely, we use the same mask to define the nucleolar region of the spread chromosomes, and obtain the intensity normalized to background (in arbitrary units) of the nucleolar Pch2 signal/cell (by excluding the signal in the masked area). We use a minimum of 30 cells (from two independent experiments) per described condition. We present the quantification of the nucleolar pool of Pch2 in Supplementary Figure 4I, and find that RNAPII-depletion has a non-significant effect on the intensity of the nucleolar Pch2 pool, which is in stark contrast to the significant effect of RNAPII-depletion of the non-nucleolar Pch2 pool (see Figure 3I). Technically, we experienced that the analysis (as further suggested by the reviewer) regarding the distribution and colocalization of Pch2 with respect to Hop1 and Gmc2 is extremely challenging (especially because the intensities of Pch2 drop significantly under depletion conditions), and feel that we cannot make any strong statements in this regard. As such, we have not included this analysis in the current manuscript. We have however now reworded our statements on the possible existence of two independent pools of Pch2 (see also point 4, reviewer 2).

*3) This same issue applies to Figures 3F, 3G, where Pch2 localization is analyzed in the orc1-161 mutant. How is Hop1 distribution/abundance in the orc1-161 mutant? Is Orc1 only required for recruitment of the transcription-associated chromosomal Pch2 pool not involved in Hop1 removal from the SC axes, or it also affects the other proposed population of Pch2 presumably acting on Hop1?*

We have now analysed the effect of the *orc1-161* mutant on Hop1 abundance on meiotic chromosomes. Strikingly, although under identical conditions we do find a significant loss of Pch2 signal, we do not detect any significant changes in the abundance of Hop1 in *orc1-161* cells (Supplementary Figure 8H). We discuss several interpretations of this observation in the revised Discussion.

*4) The authors view as “striking” the observation that Pch2 ChIP-seq peaks do not coincide with Hop1-enriched (axis) sites. Given the assumption that Pch2 removes Hop1 from chromosomes, I would consider that result as “expected”. Another question is the finding that the ATP-hydrolysis defective Pch2-E399Q version neither shows overlap with Hop1 regions; this is more surprising. As the authors explain in the manuscript, Pch2-E399Q is expected to remain locked to its client(s)/substrate(s)/adaptor(s). Indeed, according to this notion, cytological assays have shown that, in contrast to wild-type Pch2, Pch2-E399Q extensively colocalizes with Hop1 both at the rDNA and chromosomes (Herruzo et al., NAR 2016). Consistent with the prediction for a more stable interaction with the substrates, Pch2-E399Q displays stronger chromatin binding compared to Pch2 as shown in Figure S1A. The authors speculate that there are two chromosomal populations of Pch2, one associated to certain transcribed regions and another one, undetectable by ChIP, responsible for Hop1 removal. Why this second pool cannot be detected, at least in the case of Pch2-E399Q, if it remains locked to Hop1, and Hop1 is detectable? What is the direct evidence that this second population of chromosomal Pch2 does exist? Do the cytologically detectable Pch2 foci represent both populations? (Please, see points 2 and 3).*

The reviewer is correct to remark that it is more surprising that pch2-E399Q does not co-localize with axis sites. However, we do believe that also wild type Pch2 would, in principle, be expected to co-localize with its substrate Hop1. First, although Pch2 removes the large bulk of Hop1 from meiotic chromosomes, it is clear from many studies that Hop1 is never completely lost from chromosomes, even in cells that are fully synapsed (as judged by IF or ChIP-seq analysis) (for example, see [4, 12, 13]). Second, the pool of Pch2 that is removing Hop1 from a certain locus at any given moment should per definition co-localize with that pool Hop1, by virtue of the enzyme-client relationship between these two factors. One could thus theoretically expect to be able to ‘catch’ these (albeit likely transient) interactions, and by ChIP-seq these should in principle manifest as co-localization. The fact that we apparently do not see these interactions could thus indicate a technical limitation of this approach (in other words, one is left to speculate that this pool of Pch2 is not detectable by ChIP approaches). As such, we agree with the reviewer that we do not have any direct proof of the existence of multiple pools of Pch2 but we still believe that we cannot exclude the existence of such populations. Having said that, the fact that we see that the total intensities of all Pch2 foci is lower under depletion conditions (see for example Figure 3H and 4F) would argue against a specific population of Pch2 that is insensitive to RNAPII/Orc1 inactivation. We have now reworded the paragraphs in the Results and Diascussion sections where discuss the localization of Pch2 versus Rec8/Red1/Hop1 to omit the statement that this observation is surprising, and to mention the IF-based observations.

*5) The authors speculate that chromatin modifications may influence Pch2 binding to certain transcribed genes. Since Sir2 and Dot1 are the usual suspects in this context, it would be interesting to determine whether these chromatin modifiers are required for Pch2 recruitment to gene bodies.*

See also point 10, reviewer 1. We have now included a correlation matrix between Pch2 binding sites and a battery of other histone modifications for which there are good yeast ChIP-seq data sets available (from mitotic cells [8]). Interestingly, among other transcription-associated histone modifications, we find a positive correlation between Pch2 binding sites and H3K79-monomethylation. Dot1, the methyltransferase that methylates H3K79 is involved in proper Pch2 localization under certain conditions [9, 10], and the BAH domain of yeast Sir3 (which is similar to the BAH domain of Orc1) is sensitive to this modification with respect to nucleosome association [11]. This suggests that the observed binding pattern of Pch2 might be related to this particular histone modification, something that we now discuss in our revised manuscript. In addition to the correlation between Pch2 and H3K79-mono-methylation, we found that Pch2 correlates with several other marks, most notably H4K20-mono-methylation, H3K56-acetylation, H3K4-demethylation and H3S10-phosphorylation. A communality between these marks is their association with active RNAPII-transcription. The data comparing the histone modifications with Pch2 data sets is now included as Supplementary Figure 7, and we discuss these findings in the manuscript. However, due to time restrictions we have not included any further analysis of Sir2/Dot1 towards these questions, also because we feel that properly following up on any such observations would require a significant amount of additional experiments, which we feel would fall outside of the current scope of the manuscript. Nonetheless, we agree with the reviewer that analyzing these factors, and their potential connection to Pch2 and transcription will be interesting to pursue in the future.  *6) The finding that Pch2 binds to a subset of transcribed genes during meiosis, raises the possibility of a possible role for Pch2 in regulating transcription that is not addressed in the manuscript. It is curious that one of the genes bound by Pch2 is HOP1. It has been shown that global levels of the Hop1 protein are increased in the pch2 mutant (Ho and Burgess, PLoS Genetics 2011), although the underlying molecular mechanism is currently unknown. Figure 4F shows that Hop1 protein levels and Hop1 phosphorylation do not appear to be significantly affected upon Rpo21-FRB depletion, a condition that prevents Pch2 binding to transcribed genes. However, quantification and repeats of this WB analysis, including pch2 deletion as control for comparison, should be provided. Also, since some low amount of Pch2 bound to genes may remain in the Rpo1-FRB depletion assay, to definitely rule out a role for Pch2 in HOP1 transcription, HOP1 mRNA levels in the pch2 deletion mutant should be analyzed.*

We agree that analysing a potential role for Pch2 in transcriptional regulation is interesting, and we now present RNA-seq experiments in wild type and *pch2Δ* cells to assess genome-wide transcriptional effects. Strikingly, we find that deleting *PCH2* has no very little effect on genome-wide mRNA levels, an no specific effects are observed within the Pch2-associated genes, including *HOP1.* We now mention this explictly in the revised manuscript: *(‘…*For example, although Pch2 is associated with the *HOP1* gene, we found no significant difference between *HOP1* mRNA levels in ndt80pch2Δ relative to ndt80Δ cells by differential expression analysis by RNA-seq (log_2_ fold: 0,190, p-value = 0.46)…’)

*Minor points:

1) Figures 1G and 1H show that elimination of the NTD of Pch2 impairs Pch2 binding to selected target genes. Thus, the authors conclude that the NTD is required for Pch2 recruitment to transcribed gene bodies. Although, the conclusion is formally correct, it is possible that the NTD is not directly involved in recruitment, but in the formation or stability of the Pch2 hexameric complex.*

We agree with this reviewer, and we have now moved the data discussing the NTD of Pch2 into Supplementary Figure 1, and we have reworded our manuscript. See also point 9, AE.

*2) What do the asterisks indicate in Figures 2H and S6B? The rDNA? Please, explain in the legends*

Indeed, we intended to indicate the nucleolus, as judged by a lack of Gmc2 staining and morphological appearance. We have now clarified this in the Materials and Methods. *3) Evidence for functionality of the 3xFLAG tagged version of Pch2 must be provided*

We now provide evidence that the FLAG-tagged version of Pch2 is functional. First, we show that in meiosis, 3XFLAG-Pch2 can interact with Orc1, as judged by Co-IP, as was also shown for 3XHA-Pch2 ([14] [1]) (Supplementary Figure 1A). In addition, we performed a spore viability assay, making use of the observation by Burgess and co-workers that *pch2Δ rad17Δ* show synergistic effects on spore viability [15, 16]. We show that expressing 3XFLAG-Pch2 (from its endogenous promoter) rescues this synthetic effect (*i.e.* 3XFLAG-Pch2 *pch2Δ rad17Δ* behave like *rad17Δ* cells) (Supplementary Figure 1B). In addition, we note that in experiments that will be presented in another manuscript, we have found that expressing 3XFLAG-Pch2 in *pch2Δ* cells rescues the effect of Pch2 on rDNA-associated DNA break formation (Villar-Fernandez *et al.*, manuscript in revision).

*4) In the WB of Figure 1B the Pch2 protein is detected at t=0 using anti-FLAG antibodies, but not in Sup Fig S4E using anti-HA antibodies? Is that an antibody issue?*

We occasionally see expression of both HA or FLAG tagged versions of Pch2 at the start of time courses (t=0). We therefore believe that this is not a peculiarity of either tag (note that both constructs are driven by the same promoter), but rather a variability in experimental conditions (*e.g.* pre-sporulation synchrony). For clarity, we have now replaced the original WB as shown in Figure 1B, as also requested by the AE.

*5) Line 148: Supplementary Figure 1B should be 1A*

This has been changed accordingly. *6) Line 168: Supplementary Figure 1C should be 1B*

This has been changed accordingly. *7) Line 171: Supplementary Figure 1D should be 1C*

This has been changed accordingly. *8) Line 185: Supplementary Figure 1E should be 1D*

This has been changed accordingly. *9) Line 188: Supplementary Figure 1F should be 1E*

This has been changed accordingly. *10) In page 1 of Supplementary data, Supplementary Figure 1D should be 1C*

This has been changed accordingly. *11) In page 2 of Supplementary data, Supplementary Figure 1B should be 1A*

This has been changed accordingly.

*Reviewer #3: Overview.
Here the authors have used whole-genome analysis and genetics to investigate the role of the the AAA+ ATPase Pch2 during meiosis in S. cerevisiae, a widely employed model system for studying the mechanisms of recombination and chromosome segregation. The authors identify regions of enriched binding along chromosomes and draw connections to the locations of genes, which is a very different pattern of enrichment to that observed for axis-associated proteins like Rec8 and Hop1. Connections with active transcription are not convincing, and it is not clear that the point the authors are trying to make is well supported. Initial data use whole-genome maps, but most of the data is targeted Q-PCR at a small number of loci. Given the differences across loci that the NGS ChIP-seq data reveals, it is not clear how representative the Q-PCR loci are. ChIP-seq in other mutants (rather than just Q-PCR) would certainly strengthen the validity of the conclusions. From a technical standpoint, I am not convinced by the use of "Peak shape score" to plot (and analyse?) raw ChIP-seq data. Is this a quantitative measure of enrichment? Or just a measure of how "good" a peak there is in the data? I offer below a number of comments that I think will help to strengthen and improve the clarity of the presented research.

General comment.
1. The text would benefit greatly from inserting more paragraph breaks and, ideally, section headings so that the logical flow from one idea/figure to the next is clear to the reader. The current text is presented, largely, as one continuous body of text that is hard to traverse.*

We have attempted to increase the readability of the manuscript by introducing more paragraph breaks. *Specific comments.*

*1. Line 131-132. Where are the individual datasets presented? Where is the demonstration that they are highly correlated? I suggest the authors include a correlation between each of the binned datasets. Plotting, as overlays, the individual datasets along part of a chromosome may also help them make this point.*

As is customary, we have assessed the correlation between individual datasets as part of the bioinformatic pipeline, and based on standard procedures, we then pooled the individual datasets. The raw data is available at (<http://www.ncbi.nlm.nih.gov/geo/)>, under accession no. GSE144835 for ChIP-seq; Access tokenedytisgebjsjtcj.

*2. Line 135. The rationale for the manner in which data are processed and peaks are called is not well described nor justified. This leads to a number of questions that require clarification:
What does "Peak shape score", the Y-axis of plots in Fig 1C, 1D (and elsewhere) mean?
Is it actually a quantitative measure of binding, or just a measure of how well (confident?) the algorithm identified a peak?
What if most of the signal is dispersed, and the real signal doesn't inherently have peaks?
Do peaks represent the majority, or the minority, of the signal reported?
the use of a very stringent threshold for peak-calling raises alarm bells to this reviewer...how biased is the subset that are being analysed to only those regions with a particular "shape" and or "strength"?
What do the data look like if plotted as a more traditional signal/input?*

Please refer to point 2 of AEs comments. We have now re-analyzed the dataset using more conventional methods, and explain the results in detail there.

*3. Line 138. The fact that the peaks arise in only a subset of RNAP PolII genes is hardly surprising, when the thresholding used means that there are only a (relative) handful of peaks being analysed...is this a fair description of the data?*

We have reworded this section. *4. Line 140...The statement that 3% of peaks arose elsewhere is somewhat misleading. The question in this reviewer's mind is: How much of the enriched signal was present within the called peaks?*

We have reworded this section.

*5. Fig 1C. Plotting the locations of ORFs would be helpful to see a wider trend of correspondence.*

We feel that including all ORFs within the large chromosomal region depicted in Figure 1C would not be practical, and we now represent zooms of larger chromosomal regions, including ORFs that show no Pch2 binding in Figure 1D, as also suggested by this reviewer.

*6. Fig 1D. Equally, plotting a wider area than displayed in Fig 1D would aid the comparison. More importantly form this plot it is not clear what the message is. Are all these genes enriched ? (They seem to be.) Clarifying the description of these data would greatly help the reader.*

See point 5.

*7. Line 143 and Fig 1E. The analysis of correspondence with gene structure (and absenece in promoters) would be strengthened by a figure similar to 1E, but where the analysis/pileup point is the gene promoter (and/or terminator). The two plots together would help to demonstrate the enrichment within ORFS and (stated, but not demonstrated) exclusion from promoters.*

Please see Figure 1E and Figure 1F, where we now also present a tiling ChIP-qPCR analysis of two Pch2-associated ORFs (*GAP1* and *TDH3*) including oligo pairs immediately upstream and downstream of the CDS of these two genes.

*8. Labelling for Sup Fig 1 seems to have got mixed up. I think all the panels are one letter out.
Figure referencing used below is based on the submitted figure labelling, not the text.*

This has been changed accordingly. *9. Fig S1A. How many peaks were present in both samples? A correlation of the shared set of peaks would be informative to determine if, on average, the same peaks are stronger in the mutant (or variable?), or whether the global increase reported is because they represent different peaks detected in the mutant.*

For wild type Pch2, there are 437 peaks and for Pch2-E399Q there are 525 peaks. We now include the raw data for two datasets in Supplementary Table 1.

*10. Line 163. Where is the data shown to support this statement that all genes occupied by Pch2 are transcribed during meiosis? What (arbitrary) cut-off was used to determine which genes are meiotically transcribed? Is it to do with induction? Or absolute level? These data and conclusions are not currently convincing.*

All the criteria and the cut-off used to determine these parameters are now described in detail in the Material and Methods. *11. Indeed, Fig S1B, C seems to directly indicate that Pch2-bound genes span the entire spectrum of gene expression levels.*

We agree with the reviewer. *12.Moreover, Line 169, requires revision: Fig 1C is about as close to no correlation (r^2=0.0026) as one can get... Why, then does the text state that a weak correlation was detected? The authors may wish to compute the correlation they (may) observe between the same number of random values for comparison...*

We have now re-analyzed our data using a new RNA-seq dataset (also presented in our manuscript) and have plotted the same graph. We now obtain a low positive correlation between expression values and Pch2 binding scores (R^2^ = 0,3789), suggesting at least a weak relationship between transcriptional activity and Pch2 association. However, as also indicated in Supplementary Figure 1E, Pch2 binding sites show a broad distribution for transcriptional strength, suggesting the presence of additional factors that influence binding patterns. *13. Separately, is it known whether "Peak shape score" is a quantitative measure? Perhaps there is a correlation between fold enrichment and expression, but not one between "Peak shape score" and expression, for example.*

See point 1, AE. *14. Line 173-179. The experiments performed by the authors to try to rule out ChIP artefacts are good, but certainly require a more thorough summary in the main text. The authors point out where artefacts may arise, so quite simply they then need to succinctly state (in the main text) how they are able to rule them out systematically. This will strengthen, not weaken, the main text.*

A statement explaining the major analyses revolving around ChIP artefacts this is now mentioned in the Results section.

*15. Fig S3A. Minor. Is 20% EtOH, the final concentration? What concentration is the drug?
Fig S3B. Minor. I suggest placing this schematic before Fig S3A.*

We have reorganized the figure as suggested. The final concentration of EtOH is 2% and the final concentration of 1,10 phenanthroline is 100 μg/ml. This is now clarified in the materials and methods section.

*16. Line 231. It would strengthen the conclusion if the published zip1D experiments (reported to show complete loss of Pch2) were repeated alongside the new experiments reported here for RNA Pol inhibition or depletion. i.e. Is it possible that this is a lab/reagent/analysis difference rather than a real difference? If this cannot be done, a qualification can be included to this effect.*

We have now performed an immunofluorescence analysis in which we compare the reduction of Pch2 localization in *zip1Δ* with our anchor away based depletion of RNAPII (Figure 2I and Supplementary Figure 4H). We find that the depletion of RNAPII leads to a significant loss of Pch2 chromosomal recruitment, but this loss is less pronounced than the loss that is seen in *zip1Δ* cells.

*17. Line 232. It is perplexing why the authors hypothesise the effect may indicate incomplete inhibition when the data presented directly demonstrate this to be the case (Fig. 2E).*

Although we agree with the reviewer that the loss of RNAPII from chromosomes is strong, it is not complete, something that is underscored in the novel analysis we have performed in which we compare *zip1Δ* with our anchor away based depletion of RNAPII (Figure 2I and Supplementary Figure 4H; see also point 16, reviewer 3). *18. Line 250-252. Please clarify this point. In the previous sentence the fold enrichment of Pch2 is assayed around 798 annotated ARSs (nothing to do with Pch2 peaks). Why then would adjusting the p-value threshold for Pch2 peaks make any difference? Are the authors themselves clear about what analyses they have performed?*

We thank the reviewer for pointing this out and apologize for this confusion. We now omit the analysis with the adjusted p-values.
 *19. Lines 303-310 and Fig 4A. Without further description and validation, I remain sceptical about plotting data Y-axis as "Peak shape score". Is this a known quantitative value that measure enrichment of these factors? Can these data be plotted as more simple fold enrichment instead? Also, an X-Y plot of binned Pch2 enriched signal vs the other factors would help to understand if this is a global quantitative positive, negative, or wholly absent relationship.*

See point 1, AE. In addition, we now provide a genome-wide correlation plot between our Pch2 data sets and the discussed data sets for Rec8, Red1 and Hop1 (Figure 2A-B). As can be seen from this plot the Pch2 data sets are highly correlated but do not correlate with Rec8, Red1 and Hop1 patterns.

*20. Line 328. Under ndt80D conditions, isn't most Hop1 already lost on chromosomes? Is there dynamic range to observe an effect?*

We consistently observe significant amounts of Hop1 in our chromosome spreads, even in “late” G2/prophase, which is likely a reflection of the fact that Hop1 is never completely removed from chromosomes in meiotic G2/prophase. To ascertain that IF analysis is within the dynamic range also analysed Hop1 in *pch2Δ* cells, and under these conditions we find increased levels of Hop1 on chromosomes, as expected (Supplementary Figure 8A).

*Would performing anchor-away Pch2 be helpful here positive control) perhaps?*

We agree that anchor away for Pch2 would be a fantastic tool. However, due to technical issues we have been unable to successfully generate a functional *PCH2-FRB* allele. *21. Lines 329-330. This part of the sentence is confusing. Perhaps clarify that loci used for analysis of Hop1 were identified based on prior datasets.*

This has been clarified accordingly. *22. Lines 331-332. Please clarify what the hypothesis is here with respect to investigating Hop1 phosphorylation state.*

This has been clarified accordingly.

References

1.	Villar-Fernández MA Cardoso da Silva R, Pan D, Weir E, Sarembe A, Raina VB, Weir JR, Vader G. A meiosis-specific AAA+ assembly reveals repurposing of ORC during budding yeast gametogenesis. BiorXiv. 2019. doi: /10.1101/598128

2.	West AM, Rosenberg SC, Ur SN, Lehmer MK, Ye Q, Hagemann G, et al. A conserved filamentous assembly underlies the structure of the meiotic chromosome axis. eLife. 2019;8.

3.	Herruzo E, Santos B, Freire R, Carballo JA, San-Segundo PA. Characterization of Pch2 localization determinants reveals a nucleolar-independent role in the meiotic recombination checkpoint. Chromosoma. 2019.

4.	Subramanian VV, Zhu X, Markowitz TE, Vale-Silva LA, San-Segundo PA, Hollingsworth NM, et al. Persistent DNA-break potential near telomeres increases initiation of meiotic recombination on short chromosomes. Nature communications. 2019;10(1):970.

5.	Shor E, Warren CL, Tietjen J, Hou Z, Muller U, Alborelli I, et al. The origin recognition complex interacts with a subset of metabolic genes tightly linked to origins of replication. PLoS Genetics. 2009;5(12):e1000755.

6.	Kuo AJ, Song J, Cheung P, Ishibe-Murakami S, Yamazoe S, Chen JK, et al. The BAH domain of ORC1 links H4K20me2 to DNA replication licensing and Meier-Gorlin syndrome. Nature. 2012;484(7392):115-9.

7.	Smolle M, Workman JL. Transcription-associated histone modifications and cryptic transcription. Biochimica et Biophysica Acta. 2013;1829(1):84-97.

8.	Weiner A, Hsieh TH, Appleboim A, Chen HV, Rahat A, Amit I, et al. High-resolution chromatin dynamics during a yeast stress response. Molecular Cell. 2015;58(2):371-86.

9.	San-Segundo PA, Roeder GS. Role for the silencing protein Dot1 in meiotic checkpoint control. Molecular Biology of the Cell. 2000;11(10):3601-15.

10.	Ontoso D, Acosta I, van Leeuwen F, Freire R, San-Segundo PA. Dot1-dependent histone H3K79 methylation promotes activation of the Mek1 meiotic checkpoint effector kinase by regulating the Hop1 adaptor. PLoS Genetics. 2013;9(1):e1003262.

11.	Armache KJ, Garlick JD, Canzio D, Narlikar GJ, Kingston RE. Structural basis of silencing: Sir3 BAH domain in complex with a nucleosome at 3.0 A resolution. Science. 2011;334(6058):977-82.

12.	Subramanian VV, MacQueen AJ, Vader G, Shinohara M, Sanchez A, Borde V, et al. Chromosome Synapsis Alleviates Mek1-Dependent Suppression of Meiotic DNA Repair. PLoS Biology. 2016;14(2):e1002369.

13.	Herruzo E, Ontoso D, Gonzalez-Arranz S, Cavero S, Lechuga A, San-Segundo PA. The Pch2 AAA+ ATPase promotes phosphorylation of the Hop1 meiotic checkpoint adaptor in response to synaptonemal complex defects. Nucleic Acids Research. 2016;44(16):7722-41.

14.	Vader G, Blitzblau HG, Tame MA, Falk JE, Curtin L, Hochwagen A. Protection of repetitive DNA borders from self-induced meiotic instability. Nature. 2011;477(7362):115-9.

15.	Wu HY, Burgess SM. Two distinct surveillance mechanisms monitor meiotic chromosome metabolism in budding yeast. Current biology : CB. 2006;16(24):2473-9.

16.	Ho HC, Burgess SM. Pch2 acts through Xrs2 and Tel1/ATM to modulate interhomolog bias and checkpoint function during meiosis. PLoS Genetics. 2011;7(11):e1002351.
