# Supplementary material for: Active transcription and Orc1 drive chromatin association of the AAA+ ATPase Pch2 during meiotic G2/prophase
Source: PLoS Genet. 2020 Jun 22;16(6):e1008905. doi: 10.1371/journal.pgen.1008905 (PMC7332104; doi:10.1371/journal.pgen.1008905)
Supplement: Attachment — Submitted filename: SIlva_point-to-pointreply_final.docx [file pgen.1008905.s020.docx]

*Dear Gerben,

Thank you very much for submitting your Research Article entitled 'Active transcription and Orc1 drive chromatin association of the AAA+ ATPase Pch2 during meiotic G2/prophase' to PLOS Genetics. Your manuscript was fully evaluated at the editorial level and by independent peer reviewers. The reviewers appreciated the attention to an important topic but identified some aspects of the manuscript that should be improved.*

We thank the reviewers for their thoughtful comments on our manuscript, and their interest in our work. We outline our point-to-point reply to the questions that were raised below/

*Below, some commentary on the reviewers’ comments:*

1. *Reviewer 1: these should be easily addressed*

We have now addressed all these points. See also below.

2. *Reviewer 2: This reviewer had three major concerns.*
1. *Biological significance. I think that this is a concern that should be addressed in the discussion. For the difference between your conclusions and those in reference 39, I think that a bit more explicit discussion of what the differences in experimental approaches were, and why they might produce a difference in results, would be warranted. A quick read of that paper indicates that, as you say, there was very little non-nucleolar Pch2 detected (I think that this might be due to how images were thresholded, but I leave it to you to decided); also, ref 39 used a functional readout for Pch2 activity (checkpoint activation in zip1∆) that might actually be the difference between SK1 and BR, since BR zip1∆ mutants arrest and SK1 only delays. Again, I think that a more explicit discussion of this and other issues is warranted.*

We note that in reference [39] and [61], Pch2 association to the non-nucleolar in BR-strains is noted the appear quantitatively different. It is obviously hard to compare technical quantification procedures. However, under certain mutant conditions, Pch2 can be observed on euchromatin in BR (see same references). We feel that, together with the fact that certain Pch2 functions appear quantitatively different (such as the *zip1∆* arrest/delay, as mentioned), this indicates that there is a likely difference in euchromatin Pch2 association between SK1 and BR*.* We have now included a more explicit discussion of these points in the manuscript (Discussion section). *With regards to the main concern, that of biological relevance, both your data and the data in reference 39 indicate that loss of Orc1 function does not have the same phenotype as a pch2 mutant, both with regards to Hop1 distributions and checkpoint function. The question remains then as to what the biological function is of Orc1-dependent Pch2 recruitment to transcriptionally active regions, and it would be useful to include at least some thoughts as to what function that recruitment might serve.*

We have now included a more explicit discussion of these points in the Discussion.

1. *This concern involved the relationship between transcription and Orc1 and Pch2 recruitment. I agree with the reviewer that a trivial explanation would be that Orc1 is necessary for transcription of genes to which Pch2 is recruited. This should be relatively easy to address, either by looking at a couple of Pch2-marked genes, or by seeing if any data exists in the literature regarding the effect of orc1 mutants on gene expression. The question of whether or not transcription is required for Orc1 binding to these genes could, to my mind, be addressed by discussing the issue in an even-handed manner.*

We agree that this is a possibility, and we have tested this idea. We now include new data (Supplementary Data 6F), where we use RT-qPCR to measure transcription (*i.e.* mRNA levels) of two selected Pch2-associated genes (*GAP1*, and *HOP1*, compared to *PPR1* (non-Pch2 associated gene) and *ACT1* as control*)* in *orc1-161* and *ORC1* cells under restrictive temperatures. We do not find any differences in expression strength of these genes under these conditions, arguing against transcriptional effects of Orc1 which might affect association of Pch2. In the current manuscript, this is now mentioned in the Results and Discussion sections. We now also mention the possible connection between transcription and Orc1 in the discussion.

*The reviewer had concerns about the apparent contradiction between the correlation between Pch2 and H3K79me1, on one hand, and biological evidence that Dot1 limits Pch2 localization to euchromatin. Given the rather weak nature of the Pch2-H3K70me correlation, I think that it remains to be shown if this correlation reflects a direct or indirect effect, and I think that it would be wise to somewhat moderate the conclusions in this section.*

We agree with this point and have moderated our conclusions in this section.

2. *Reviewer 3: All the concerns of this reviewer need to be addressed, but it appears to me that most can readily be addressed by providing additional information in the text or in supplement without additional experimentation. However, I do wish to amplify the reviewer’s point regarding Supplementary Figure 1F, where it is abundantly clear that the regression line is being driven almost entirely by the points with expression values of ~0.8, and that removal of this subset would result in no correlation at all. In light of this, I would suggest that the conclusions be altered to say that transcriptional strength is not a factor that determines Pch2 binding.*

We agree that there is a weak correlation between expression strength. We removed the subset of genes (surrounding expression levels of ~0.8) that were predicted by the reviewer to potentially lead to a weaker correlation scenario and re-plotted both correlations (see also the answer to point 8, reviewer 3, and **Figure C** below). We found a subtle decrease in the Pearson correlation coefficient in this data relative to the original dataset (that, for completeness, we have chosen to still present in the manuscript and Supplementary Figure 2). Thus, transcriptional strength is likely not the sole factor dictating Pch2 binding. We emphasize this in the manuscript stating “transcriptional strength *per se* likely plays, if any, only a minor role in dictating Pch2 binding”.

*Finally, this reviewer suggested (in their first point #1)(!) dividing the text further into paragraphs. I agree that this will increase readability and suggest the following lines as dividing points, to be divided after the full stop unless otherwise indicated. It would help if paragraphs were indented throughout the manuscript.*

*Line 134, at start
Line 154, at start
Line 170
Line 237
Line 257
Line 315*

These paragraph changes have been included.

*Also, please consider the following:
Line 331, suggest, “we provide evidence below”*

This has been changed.

*Line 395, suggests “also transcriptionally active”*

This has been changed.

*Line 529-30, redundant with Lines 525-6*

This has been changed.

*Throughout Materials and Methods, please provide antibody dilutions used.*

This has been included.

*References—improved, but journal abbreviations are still inconsistent.*

This has been corrected.

*Figures—for ChIP-seq graphs, please indicate bin sizes and provide units for “normalized reads” (i.e. such as reads per million per 10kb) in either the figure itself or the legend.*

In the legends of the respective figures, we now mention: “Genome browser view representative images (RPKM; see also Material and Methods)”. In the Material and Methods (section Processing of ChIP-seq) we state: “data visualized on IGV [74] were normalized to the number of Reads per Kilobase per Million (RPKM) of total mapped reads. Bin size was set to 25”.

*Reviewer #1: The authors have done a good job addressing the most pressing and substantive questions and concerns brought up by all the reviewers. While it's still a mystery why Pch2 localizes to a subset of transcribed genes in meiosis, I am convinced that it does indeed do so. I just have a couple of minor points for the authors to address before acceptance:*

*Phenanthroline is mis-spelled twice, I believe.*

This has been corrected. *Figure 2B - labels along the top are in the opposite order from what they should be.*

This has been corrected. *Figure 6E - it’s a little confusing to have “zip1d” on a different line than “3XFLAG-Pch2-E399Q” - can the authors move it to the same line?*

This has been corrected.

*Reviewer #2: In this revised version of the manuscript by Cardoso de Silva et al. the authors have performed additional work and they have satisfactorily addressed and/or responded to most of the concerns raised to the previous version. However, there are still some issues that, in my opinion, remain to be solved.*

***1.*** *Although the discussion regarding the possible existence of different populations of Pch2 with different functions (one presumably undetectable by ChIP-seq affecting Hop1 and another one detectable by ChIP-seq with no effect on Hop1, but both of them cytologically detectable) has been softened and additional possibilities are now mentioned, my main concern with this article still is the lack of some biological relevance for the transcription-dependent binding of Pch2 to chromosomes.*

We agree that one of the main questions that remains based on our current data is the biological relevance of the Pch2-binding profiles. While we show that the binding is determined by active transcription and Orc1, we still do not know what Pch2 is doing at these sites. We now discuss potential roles in the conclusions, also connected to the known connection with Orc1, as described by us and others. See also point 2, editor ML.

***2.*** *The authors now succinctly present some ChIP-seq data on the binding of Pch2 to the rDNA region describing that it binds to the RNAPI-transcribed 25S region. Since Orc1 is also required for Pch2 nucleolar targeting there could be also a connection between Orc1 and transcription by RNAPI in the rDNA. I agree that the detailed characterization of the Pch2 rDNA targeting is beyond the scope of this paper, but the possible connection between Orc1 and transcription on the “euchromatin” could be further explored. This paper describes that Pch2 recruitment to chromosomes requires Orc1 and requires RNAPII transcription, but the dependence/relationship between Orc1 and transcription is not analyzed. In other words, is Orc1 required for transcription at the ORFs bound by Pch2? Is transcription required for Orc1 binding to those ORFs? I don’t really like bringing up new issues during revision, but the results presented in the revised version lead to these questions that should be addressed or, at least, further discussed.*

We agree that this is a possibility, and we have tested this idea. We now include new data (Supplementary Data 6F), where we use RT-qPCR to measure transcription (*i.e.* mRNA levels) of two selected Pch2-associated genes (*GAP1*, and *HOP1*, compared to *PPR1* (non-Pch2 associated gene) and *ACT1* as control*)* in *orc1-161* and *ORC1* cells under restrictive temperatures. We do not find any differences in expression strength of these genes under these conditions, arguing against transcriptional effects of Orc1 which might affect association of Pch2. In the current manuscript, this is now mentioned in the Results and Discussion sections. We now also mention the possible connection between transcription and Orc1 in the discussion.

See also response to point 2, editor ML.

***3.*** *The authors now present a correlation analysis between genomic distribution of Pch2 and a panel of histone modifications obtained from mitotic cells. They comment on a certain degree of correlation between Pch2 and H3K79me1. However, previous cytological analyses (Ref. 23) go in the opposite direction because Dot1-dependent H3K79 methylation appears to prevent Pch2 mislocalization to euchromatin. That study shows that Pch2 mislocalizes outside the rDNA in dot1 and H3K79 mutants. The authors have opted for not directly analyzing transcription-dependent Pch2 binding in dot1 (and sir2) mutants arguing time restrictions. Although genome-wide ChIP-seq studies could take more time, at least ChIP-qPCR analyses at the same selected target genes explored in other experiments could have been done.*

We agree with this reviewer that pursuing these questions further is an interesting avenue. However, we believe that these experiments currently fall beyond the scope of this manuscript. We do now expand the discussion of these points in the text, as also suggested by the editor.

*Minor points:

Line 245: Supplementary Figure 3E should be 4E*

This has been corrected. *Line 270: This (delete “is”) observation is in agreement…*

This has been corrected. *Lines 390-392 and Supplementary Figure 8G, 8H. Has been the nucleolus included in the quantification of Hop1 “chromosomal” levels? Ref 39 shows that the absence of Pch2 in the nucleolus in orc1 mutants leads to Hop1 mistargeting to the rDNA region. Please, mark the nucleolus in the spread nucleus displayed in Fig S8G to show that Hop1 is mistargeted to the rDNA in orc1-161. The nucleolar area must be eliminated for accurate Hop1 chromosomal quantification.*

We determined the location of the nucleolus by checking the DNA signal (DAPI) not co-stained with Gmc2, a marker for the synaptonemal complex. As expected, Hop1 was found within this region in *orc1-16*1 strains but not in *ORC1* strains in all the data set analysed. We have substituted the Supplementary Figure 9G. Furthermore, Hop1 level was re-quantitated by excluding the Hop1 signal outside the Gcm2 surface (green channel). Supplementary Figure 9H was modified accordingly. We re-quantitated the data presented on Figures 5B and S9C.

*Reviewer #3:* (please note that, for clarity, point numbering is added/altered as compared to original report)

*The authors have made some modest improvements to the presentation of their work. In particular, they have replaced the use of Peak shape score with a more simple presentation of ChIP-seq reads per million. Nevertheless, a number of outstanding queries arise regarding the calculation and presentation of data using this new metric (specific comments below), plus a number of points that were raised during the first round of revision but have not been answered or addressed.*

*General comment:*

***1****. Despite the author’s response, I still find the text to have unnecessarily large blocks of text, hampering readability and therefore critical understanding. For example, the entire introduction (2 pages, has only 4 paragraphs). Within the results, there is not a single paragraph break on page 5 nor on page 8, and only one on each of pages 10-13. These sections cover and discuss a lot of complex data, frequently pointing between main and supplementary figures. Giving the text (and the reader!) more space to breathe and take stock of what is being presented in each section of results would really help.*

We apologize to the reviewer for this oversight. We have now included paragraph breaks in the text, as suggested. See also point 3, editor (ML).

*Specific comments:*

***1.*** *Line 139-141. We respectfully request (again) that the correlation between repeat datasets are presented in the manuscript. The methods (Line 624) indicate that a program: multiBAMsummary (v 1.2) was used. Please describe what quantitative correlation this software measures, what bin size was used (and justify its use), and then state the pairwise results between the replicate datasets. I note that the default bin size is 10 kb (https://deeptools.readthedocs.io/en/develop/content/tools/multiBamSummary.html), which seems inappropriate for these data.*

We have added the pairwise correlation (Spearman) comparing the signal of three replicates (ChIP and Input data). The analyses indicate reproducibility across biological replicates (please see Supplementary Figure 2A and B). We employed the Spearman pairwise correlation in all our analyses. Indeed, the default bin size (10Kb) is not applicable for our datasets and we mistakenly stated the use of default parameter in the manuscript. We thank the reviewer for spotting this. We had set the bin size at every 200 bp (larger than the average contig length of our dataset) to favor a better resolution. These parameters are now better described in Material and Methods (section Computational analysis).

*2. Although it may be standard practice to plot/compute the correlation between binned data in this way, if much of the read data are noise (i.e. relatively uniform), which is quite likely in ChIP-seq data, it may make more sense to compute the correlation only between the signals present within the called peaks, or to threshold the data first so that all the low occupancy (noisy) bins are ignored in the correlation.*

*Moreover, was the same number of peaks called in every dataset? Were they in the same position?*

*Finally, as previously suggested, presenting replicate datasets as overlays (to demonstrate whether or not each sample has a relatively similar profile pattern) would be a visual way help convince the reader that what is being presented is both reproducible and meaningful.*

As described in Materials and Methods (section Computational Analysis), we limited our read coverage analyses specifically for Pch2 binding sites by creating a BED file encompassing the Pch2 binding sites (see section Computational Analysis).

As described in Materials and Methods (section Computational Analysis), the different ChIP-seq replicas were pooled after calling the peaks and the described high confidence set peaks are shared among pooled peaks. We now present a table with the number of peaks as well as the proportion of peaks of each replica found in the pooled data, which further confirms the reproducibility of the data (See supplementary Table 3).

Given that the estimation of the correlation between ChIP replicas was performed (Supplementary Figure 2A and B), we believe that the high correlation observed between replicas show that the data is reproducible. In fact, computing the ChIP-seq enrichment profiles of Pch2 (log2 ratio) of three different replicas shows signals with similar trends relative to a plot presented in the manuscript (see Figure 1E and see below for this plot (**Figure A**)). We believe that the ratio plots encompassing the pooled datasets presented in the manuscript provide a good representation of the Pch2 binding sites.

**Figure A. Enrichment profile of Pch2-E399Q occupancy in three independent replicas, as show for the averaged data set in Figure 1E.**

***3.*** *I appreciate the replacement of the Peak shape score metric with one that is more a direct measure of occupancy at each location. However, this metric is still not well described. What are the units for example? I assume it is reads/million mapped? But is this per bp, or per kb, or something else? Further: how is this metric affected by the “normalisation against input”. How was this done? Were data binned and then divided? At what bin-size? Is the final value therefore a ratio? If so, should the data be plotted on a log scale of enrichment similar to other plots in the paper? (e.g. Fig 1E). This information needs to be added to the methods and summarised in the legend/figure. Moreover, why have the “input” controls profiles been removed from the revision? They are valuable information because they enable the reader to appreciate the inherent “peakyness” (or not) of the input relative to the actual quantified data.*

We apologize for the lack of clarity in our descriptions. We normalized the reads using RPKM (Reads Per Kilobase per Million mapped reads) (see Material and Methods (section Processing of ChIP-seq), and defined the bin size at every 25 bp to secure a high resolution. Similar parameters were previously employed for visualization of Chip-seq datasets of meiotic proteins [1]. Details are now added to Material and Methods section. Peaks (coverage tracks of Pch2, Hop1, Red1 and Rec8) displayed in the manuscript were normalized as above. We have now corrected the figure legends and again apologize for the confusion this might have raised. Input plots used for ChIP-seq peak normalization were added to the manuscript in Supplementary Figure 1D.

*One of our question has not been answered, and still stands: “What if most of the signal is dispersed?…Do peaks represent the majority, or the minority, of the signal reported?”*

See answer to point 4 below.

***4.*** *The fact that 98% of called peaks overlap RNAPolI genes sounds convincing. However, (as above):* ***A)*** *what fraction of the total ChIP-seq reads did this represent?* ***B)*** *What is the expected (random) overlap given the average width of the called peaks and the fraction of the genome that is an RNA Pol II gene? More significantly, given that the ChIP-seq signal can arise via an indirect crosslink between the antigen and a histone (within the genic regions) and the DNA, it might be expected that signal would preferentially arise within gene bodies where histones have greater occupancy levels. Perhaps comparing the distribution of Pch2 to that of other factors (that don’t enrich in gene bodies) would help make the distinction here. In fact – an overlay on Fig 1D of Hop1/Red1 etc would really be useful in this regard because it is the only figure that shows the relationship between the ChIP-seq profiles and gene structure, which itself is a major finding of the study.*

**A)** We analyzed the global enrichment of ChIP and Input data with the aid of the fingerprinting plot tool Signal Extraction Scale (SES) prior to performing downstream analyses on our ChIP-seq data. SES computes the number of reads in non-overlapping partitions and gives the proportion of reads in each window. A more equal and uniform distribution of reads among equally sized genomic partitions between ChIP and Input would point to a low and spread signal of ChIP. As expected, given the presented ChIP-seq data, our analyses indicated that this is not the case. The plot (**Figure B**) shown below depicts the rightward deflection of the Pch2-E399Q ChIP dataset curve. The Input signal followed, as expected, a different trend. Analysis of the point of intersection from the axes of the ChIP signal indicates that a smaller proportion of the signal was restricted to a smaller proportion of bins. This indicates that the Pch2 peaks represent the majority of the signal presented.

**Figure B - Fingerprinting plot (deeptools, 100bp bins) displaying the cumulative percent of total reads found in a given percent of the yeast genome.**

**B)** We used the shuffleBed, BEDTools v2.16.2 tool to randomize the Pch2 binding sites to 1000 sets (binding exclusively to RNA-Pol II genes). We then intersected the Pch2 binding sites with the randomized Pch2 binding sites to determine if both sets overlap more than it would be expected by chance. We found an overlap of 9 sites between 436 Pch2 binding sites and 436 shuffled binding sites. We further computed the probability of obtaining the observed overlap between Pch2 peaks within random Pol II genes using the BEDTools Fisher’s exact test *p*-value under the null hypothesis of overlaps occurring at random, which was *p*=0.0037. These analyses suggest that binding of Pch2 to the defined sites were not a consequence of random occurrence.

**C)** We now present a genome browser high-resolution view of Hop1-ChIP of the Pch2 binding patterns shown in Figure 1D in Supplementary Figure 1E, which follows a different binding pattern relative to Pch2 (Supplementary Figure 1E).

***5.*** *Supplying the raw data is important, but supplying a large table is not very helpful for the average reader. Moreover, my original query remains answered: “A correlation of the shared set of peaks would be informative to determine if, on average, the same peaks are stronger in the mutant (or variable?), or whether the global increase reported is because they represent different peaks detected in the mutant.*

We plotted the pairwise correlation between the strength of shared peaks between Pch2 wild type and E399Q. The analyses indicate that the increased signal observed in E399Q binding relative to wild type is not originated from the additional peaks exclusively detected in the Pch2-E399Q dataset. Please refer to Supplementary Figure 2C.

*6.“ Line 166-169. It is (still) not clear where the data that underpin this statement is presented in the manuscript. This is an important point in the text. Why are there no figures to support this statement?*

We have plotted the average distribution of normalized RNA-seq counts (TPM, transcripts per million) from *ndt80Δ* and sub-plotted the distribution of mRNA expression of the Pch2 binding genes (wild-type), which entirely occupies the pool of actively expressed genes (Supplementary Figure S11C). The established cut-off is described in Materials and Methods, section RNA-seq data analysis.

*7. The addition of Fig 1F (And associated description in the text is welcomed). I would only ask for Fig 1F to be plotted in a square form such that the total spread of the dats in the X and Y directions are similar. As presented this is far from the case, with no apparent justification.*

The figure was changed accordingly.

*8. The authors may also wish to consider that these raw data actually reveal two clusters of data (zero/noise expression and non zero/real expression)—where neither display a quantitative correlation with Pch2 occupancy as measured by RNA-seq and ChIP-seq:*

*I leave it for the authors to decide whether or not to make any changes to their interpretations.*

We thank the reviewer for pointing this out. We agree that the correlation is weak and we agree that this indicates that transcriptional strength is not a strong driver of association. As suggested, we removed the subset of genes (surrounding expression levels of ~0.8) that were predicted by the reviewer to potentially lead to a weaker correlation scenario and re-plotted both correlations (Compare left and right plots in **Figure C** below). We found a subtle decrease in the Pearson correlation coefficient in this data relative to the original dataset (*i.e.* R^2^ changes from 0.3789 to 0.3726). Because we feel that presented the correlation using the entire dataset is most complete, we have kept the original plot in the manuscript but emphasize in the manuscript that this is not a strong correlation, which emphasizes the idea that transcription strength is not a strong factor dictating binding of Pch2 to these sites. See also answer to point 3, editor ML).

**Figure C. Comparison of correlation values between expression strength and ChIP enrichment**

***9.*** *Do the authors mean to refer to Fig 3I in their response? If so, where do the data indicate that the loss in zip1 ndt80 is greater than the loss in the anchor-away system? This difference does not appear to have been tested statistically. Interpretation here is unclear: do the authors consider that this difference is important?*

We have now added the statistics between DMSO treatment and *ndt80Δ* and between Rapamycin treatment and *zip1Δ ndt80Δ* in Figure 3I. The statistical analysis indicates the Pch2 levels in *zip1Δ* are significantly more affected than under Rpo21-depleted conditions.

***10.*** *It would make more sense to describe the results as demonstrating that not only was there no enrichment of Pch2 around ARSs, that ARSs actually displayed local depletion for Pch2 binding (since this is the most obvious feature of the plot presented in Fig 4B).*

We have now included a statement on this in the manuscript.

***11.*** *Line 973 and Fig 2B. Something weird has gone on with the labelling here. I think the strains on the X-axis (top) are in the reverse order. Please correct.*

The figure was corrected.

*12.Line 973. Should this say Pearson or Spearman…? Please describe how these correlations were calculated. Were the data binned before analysis? At what resolution? Were data thresholded first?*

We apologize for the confusion: it should read Spearman. We have corrected the legend. Parameters for pairwise correlation are detailed in Materials and Methods (section Computational Analysis).

***13.*** *The rewording (lines 378-381), is still very hard to follow. I suggest to replace: “based on published Hop1 ChIP-seq datasets in wild type and pch2∆ cells [44]” with “around a known Hop1-binding site [44]…”.*

The text was modified accordingly.

**References**

1.	Subramanian VV, Zhu X, Markowitz TE, Vale-Silva LA, San-Segundo PA, Hollingsworth NM, et al. Persistent DNA-break potential near telomeres increases initiation of meiotic recombination on short chromosomes. Nature Communications. 2019;10(1):970.
